# Impact of Glaucomatous Ganglion Cell Damage on Central Visual Function

**DOI:** 10.1146/annurev-vision-110223-123044

**Published:** 2024-09

**Authors:** MiYoung Kwon

**Affiliations:** Department of Psychology, Northeastern University, Boston, Massachusetts, USA

**Keywords:** glaucoma, retinal ganglion cells, contrast sensitivity, reading, spatial summation, cortical magnification

## Abstract

Glaucoma, a leading cause of irreversible blindness, is characterized by the progressive loss of retinal ganglion cells (RGCs) and subsequent visual field defects. RGCs, as the final output neurons of the retina, perform key computations underpinning human pattern vision, such as contrast coding. Conventionally, glaucoma has been associated with peripheral vision loss, and thus, relatively little attention has been paid to deficits in central vision. However, recent advancements in retinal imaging techniques have significantly bolstered research into glaucomatous damage of the macula, revealing that it is prevalent even in the early stages of glaucoma. Thus, it is an opportune time to explore how glaucomatous damage undermines the perceptual processes associated with central visual function. This review showcases recent studies addressing central dysfunction in the early and moderate stages of glaucoma. It further emphasizes the need to characterize glaucomatous damage in both central and peripheral vision, as they jointly affect an individual’s everyday activities.

## 1. INTRODUCTION

Situated in the innermost layer of the retina, retinal ganglion cells (RGCs) are the ultimate output neurons of the retina, where the initial encoding of visual sensory information into spikes takes place. RGCs thus serve as a bridge connecting the retina and the visual brain. However, the role of RGCs goes beyond merely transmitting visual signals to the brain. They also play a crucial role in encoding a range of visual features required for human pattern vision, such as contrast, color, motion, and fine or coarse textures (Sanes & Masland 2015). RGCs, thus, inarguably make up a fundamental building block of human visual processing and visual perception.

For this reason, damage to the axons or cell bodies of RGCs can lead to devastating visual dysfunction or even blindness. Glaucoma is a leading cause of irreversible blindness worldwide, characterized by the progressive loss of RGCs and their respective axons (i.e., structural and neural damage) and associated visual field defects (i.e., functional defects). Both the diagnosis and monitoring of glaucoma are based on ophthalmic testing to identify the pattern of structural changes and functional vision loss (Harwerth et al. 2010). Loss or dysfunction of RGCs has been well established in animal models of experimentally induced glaucoma, where RGC counts in the retinas of rats or monkeys were evaluated after periods of elevated intraocular pressure (Harwerth et al. 2004, Quigley & Addicks 1980, Sellés-Navarro et al. 1996). Similarly, histological investigations of human glaucomatous eyes have also confirmed the correlation between the number of ganglion cell bodies or axons and perimetric sensitivity (Garway-Heath et al. 2000, Kerrigan-Baumrind et al. 2000, Quigley et al. 1989). Therefore, glaucoma provides the unique opportunity to probe the extent and nature of the impact of RGC loss on human pattern vision.

In this review, I explore the functional consequences of glaucomatous ganglion cell damage for central visual function. Primary open angle glaucoma, the most common type of glaucoma, is conventionally associated with a pattern of peripheral visual field loss followed by central visual field loss in later stages of the disease. As such, central vision has been thought to be preserved until the end stages of the disease. This view largely stems from the use of perimetry tests optimized for the detection of peripheral vision loss, such as the Humphrey Field Analyzer (HFA) 24–2 test and high-contrast Snellen-type acuity measurements for evaluating central vision (Stamper 1989). Such functional measurements often result in an overestimation of peripheral vision loss and an underestimation of central vision loss. In addition, glaucoma is typically diagnosed by evaluating optic nerve head (ONH) damage (e.g., the dimensions of the ONH cup and disc) via optical coherence tomography (OCT). Consequently, macular damage or deficits in central vision may be easily overlooked or not fully recognized.

The conventional view of glaucoma has been challenged by recent anatomical and psychophysical evidence collectively supporting the presence of significant macular damage and central visual dysfunctions. For example, it has been shown that, even in the early and moderate stages of glaucoma, there is a loss of RGCs and shrinkage in the dendritic structures of remaining cells (Hood et al. 2013). In alignment with this structural damage, significant dysfunctions have also been observed in tasks related to central vision, such as reading, and face or object recognition (Burton et al. 2012, Glen et al. 2013, Goddin et al. 2023, Ikeda et al. 2021, Issashar Leibovitzh et al. 2023, Kwon et al. 2017, Lenoble et al. 2016, Mathews et al. 2015, Ramulu et al. 2013, Roux-Sibilon et al. 2018, Smith et al. 2014, Sohail et al. 2023, Stievenard et al. 2021). In fact, this challenge to the conventional view is not new; it dates back to at least the 1960s and 1980s, when vision researchers like Duke-Elder and Stamper brought attention to the presence of compromised central visual function even in the early stages of glaucoma, including in color vision, contrast sensitivity, and macular light sensitivity (Stamper 1984, 1989), along with frequent complaints from patients regarding problems with their central vision (Duke-Elder 1969). With recent advancements in retinal imaging techniques, such as OCT, a growing body of imaging studies has provided compelling evidence of early macular damage in glaucoma (Hood et al. 2013). Thus, there is a need for a better understanding of how such macular damage impacts central visual function in individuals with glaucoma. Approximately 50% of RGCs are located within a 4.5-mm radius of the fovea, corresponding to the central ±8° visual field, even though this macular region constitutes just 7.3% of the total retinal area (Curcio & Allen 1990). The macula plays a crucial role in various everyday visual functions; therefore, damage to it significantly impacts the quality of life for individuals with glaucoma (McKean-Cowdin et al. 2008).

A wide range of visual deficits are associated with glaucoma, including deficits in motion perception (Bosworth et al. 1997, Bullimore et al. 1993, Joffe et al. 1997), visual search (Smith et al. 2011, Wiecek et al. 2012), driving (Haymes et al. 2008, Kwon et al. 2016, Lee et al. 2018, Wood et al. 2016), eye–hand coordination (Kotecha et al. 2009, Zwierko et al. 2019), mobility (Friedman et al. 2007, Turano 1999), and gait (Bicket et al. 2020, E et al. 2021, Mihailovic et al. 2017). In this review, however, I mainly focus on recent research related to central pattern vision in the early and moderate stages of glaucoma, an area that has received relatively little attention. Note that the term pattern vision typically refers to visual perception of stationary, two-dimensional luminance patterns. In this review, the terms spatial vision, pattern vision, and pattern perception are used interchangeably. This review also provides insights into spatial visual processing following ganglion cell loss and is structured around three key components: (*a*) linking RGCs to human pattern vision, (*b*) glaucomatous RGC damage in the macula, and (*c*) functional consequences of glaucomatous damage for central visual function.

## 2. LINKING RETINAL GANGLION CELLS TO HUMAN PATTERN VISION

Being able to see and recognize the world around us requires the transmission of visual sensory information from the eye to the cortex. The human retina consists of several layers of retinal neurons interconnected by synapses and is supported by an outer layer of pigmented epithelial cells. When light enters the eye, it stimulates photoreceptors situated in the outer layer of the retina, converting light into electrical signals. These signals are then conveyed through other neurons—horizontal, bipolar, and amacrine cells—to RGCs. RGCs, located in the innermost retinal layer, transmit visual information to the visual brain via their axons. However, rather than acting as a simple relay of retinal signals to downstream cortical areas, RGCs play an active role in extracting visual features, such as contrast, edges or contours, color, and motion (Sanes & Masland 2015), and are also involved in gain control through recurrent circuitry feedback to the inner retina (Vlasiuk & Asari 2021). Moreover, RGCs represent the first stage in which visual sensory information is encoded into spikes, thereby setting an upper limit on cortical visual processing. Thus, seemingly disparate perceptual asymmetries, such as upper–lower visual field asymmetry, eccentricity-dependent visual sensitivity, and the contrast polarity effect (i.e., the difference in sensitivity between black-on-white versus white-on-black targets), have been shown to trace back to the inherent characteristics of ganglion cell neurons (e.g., asymmetries between ON and OFF pathways, as well as the nonuniform distribution of ganglion cell density across the retina) (Kwon & Liu 2019; Pons et al. 2017; Ratliff et al. 2010; Redmond et al. 2010b; Rovamo 1978; Vassilev et al. 2005; Volbrecht et al. 2000a,b; Watson 1987; Zaghloul et al. 2003).

In this section, I briefly review the theoretical foundations that may explain how the loss or damage of RGCs undermines central pattern vision, before delving into the significant structural and functional changes observed in the macula associated with glaucoma.

### 2.1. Differentiation and Convergence of Visual Information

Integration of visual sensory information over space and time is critical for constructing a coherent and meaningful representation of the visual world around us, enabling us to recognize visual patterns, perceive objects and scenes, and detect motion. It is well established that RGCs play a crucial role in both spatial and temporal integration during the early stages of visual processing (Barlow 1953, Enroth-Cugell & Robson 1966, Freed & Sterling 1988). The integration process involves two distinct yet complementary mechanisms, differentiation (e.g., lateral inhibition and surround suppression) and convergence, which occur at various processing stages along the visual pathway.

Lateral inhibition, primarily occurring at the level of the retina, refines sensory information (e.g., enhancing edge detection) by inhibiting the spread of action potentials from excited neurons to neighboring neurons (Byrne 1997, Yantis 2014). Surround suppression, which is akin to lateral inhibition, helps enhance relevant visual information by moderating a neuron’s response to surrounding or neighboring stimuli within its primary receptive field, thereby reducing its responsiveness to less relevant information (Smith 2006). Lateral connections within the early visual cortex (V1), as well as cortical feedback from higher areas, are known to play a role in surround suppression (Cavanaugh et al. 2002, Sullivan & de Sa 2006). On the other hand, convergence allows a neuron to receive input from many neurons in a network. As a result of convergence, the receptive field of each neuron increases in size with each successive level of the synaptic relay. Thus, the combined processes of differentiation and convergence enable the visual system to respond to increasingly complex types of visual signals, from light detection to pattern recognition.

### 2.2. Contrast Coding via the Center-Surround Receptive Field Structure of Retinal Ganglion Cells

**Figure 1:**
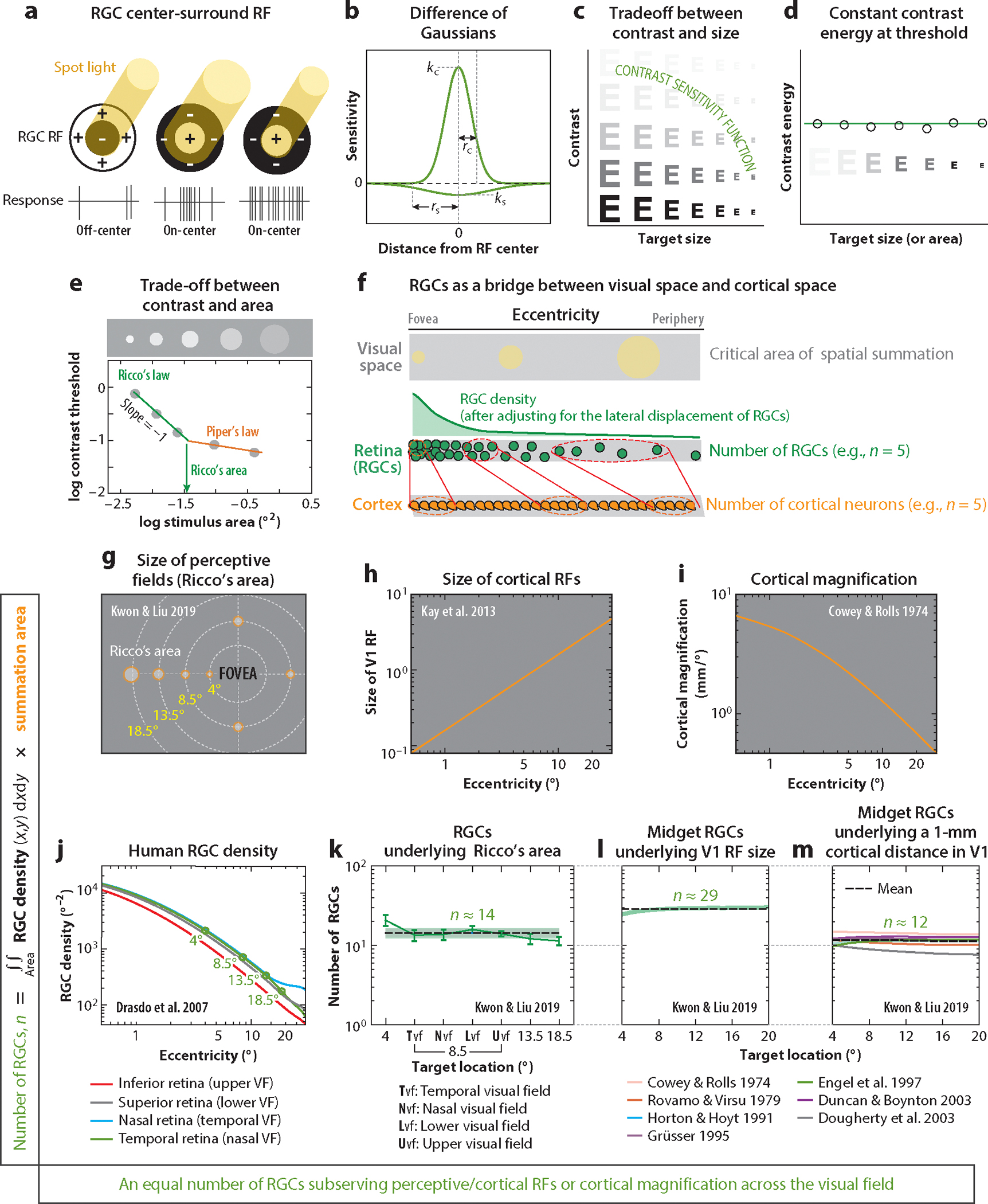
Retinal ganglion cells (RGCs) linking the retina to the cortex. (*a*) Contrast information coded by the center-surround receptive field (RF) structure of RGCs. (*b*) The difference of Gaussians (DoGs) used to model a neuron’s contrast response through the RGC center-surround receptive field. (*c*) The contrast sensitivity function (CSF) reflecting the trade-off between threshold contrast and target size. (*d*) The constant contrast energy at the threshold. (*e*) The inverse relationship between the threshold contrast and the target area, characterized by Ricco’s law and Piper’s law. (*f*) RGCs connecting visual space and cortical space. (*g*) The size of perceptive fields (Ricco’s area) varying across the visual field. For visualization purposes, the size of Ricco’s area has been enlarged proportionally to illustrate this variation. Data are sourced from Kwon & Liu (2019). (*h*) The size of cortical receptive fields as a function of eccentricity (°). Data are sourced from Kay et al. (2013). (*i*) Cortical magnification (mm/°) as a function of eccentricity (°). Data are sourced from Cowey & Rolls (1974). (*j*) The human RGC density (°^−2^) as a function of eccentricity (°) for the four meridians. Data are sourced from Drasdo et al. (2007). (*k*) An equal number of RGCs subserving perceptive RFs (Ricco’s area) across the visual field. (*l*) An equal number of midget RGCs (mRGCs) subserving the size of a V1 RF (diameter in degrees) as a function of eccentricity. The size of a population RF (pRF) in V1 is based on the data provided by Kay et al.’s (2013) functional magnetic resonance imaging (fMRI) study on humans. (*m*) An equal number of mRGCs underlying a 1-mm cortical distance in V1. The estimation is based on the human V1 cortical magnification factor data obtained from previous studies (Cowey & Rolls 1974, Dougherty et al. 2003, Duncan & Boynton 2003, Engel et al. 1997, Grüsser 1995, Horton & Hoyt 1991, Rovamo & Virsu 1979). Solid lines indicate the number of mRGCs estimated from each study. The dashed black line denotes the average value collapsed across different studies. Plots in panels *k–m* adapted from Kwon & Liu (2019) (CC BY-NC-ND 4.0).

Luminance contrast is defined as the difference in intensity between light and dark areas within an image. The threshold contrast refers to the minimum amount of contrast required for a person to detect a target (such as a spot of light or other luminance pattern) with a given probability (Figure 1c). It is important to note that contrast, rather than light intensity, serves as the primary signal sent from the eye into the primary visual cortex, forming the fundamental basis for human pattern vision.

The sensory computations involved in enhancing image contrast primarily take place in the early stages of the human visual system, particularly in the retina and along the visual pathways from the retina to the striate cortex (V1) via the lateral geniculate nucleus of the thalamus. Lateral inhibition is known as the key mechanism underlying the enhancement of spatial contrasts in objects (Marr & Hildreth 1980, Turner et al. 2018). In the retina, lateral inhibition occurs when interneurons, such as horizontal and amacrine cells, pool signals from neighboring presynaptic feedforward cells, such as photoreceptors and bipolar cells, and subsequently send inhibitory signals back to them (Gilbert et al. 1990). As illustrated in Figure 1a, the classic center-surround receptive field (RF) structure of a ganglion cell embodies lateral inhibition in the retina via the combination of the excitatory center formed by the feedforward cells and the inhibitory surround formed by the interneurons (Turner et al. 2018). Thus, contrast information is first encoded by the center and surround RF structure of a ganglion cell, typically modeled as a difference of Gaussians (Barlow 1953), as depicted in Figure 1b. This contrast information is further processed in the downstream cortical areas. When the visual system cannot receive or process the full range of contrast signals, it can have significant adverse effects on various daily activities (Owsley 2003), including reading (Leat & Woodhouse 1993), object or face recognition (Avidan et al. 2002), visual search (Paulun et al. 2015), walking (Moes & Lombardi 2009), and driving (Kwon et al. 2016, Owsley et al. 2001). For this reason, contrast sensitivity, which is the reciprocal of threshold contrast, is considered a critical metric for assessing human visual function (Pelli & Bex 2013). Given the central role of RGCs in contrast coding, and the fact that the loss of RGCs is a defining characteristic of glaucoma, it is not surprising that contrast sensitivity serves as the basis for ophthalmic testing, such as standard perimetry, to evaluate the extent and pattern of visual field loss in glaucoma.

### 2.3. The Inverse Relationship Between Threshold Contrast and Target Size (or Summation Area)

Although contrast sensitivity (i.e., 1/threshold contrast) is a commonly used functional measure, its connection to the size or area of a target is often overlooked. However, this inverse relationship is particularly relevant to evaluating the visual function of individuals with visual impairments.

As demonstrated in Figure 1c, it is apparent that large letters are more visible than small letters at near-threshold contrasts. Likewise, for near-threshold sizes, high-contrast letters are more discernible compared to low-contrast letters. This trade-off occurs because visual detection or recognition of a target requires integrating contrast energy over the target area until it reaches the threshold value.

This inverse relationship is well described by both Ricco’s law and Piper’s law. According to Ricco’s law, when small targets are projected on the retina, the total contrast energy of the target stimulus is constant at the threshold (Figure 1d). Thus, the product of the area (*A*) and intensity (*I*) of the target stimulus remains constant (*k* = *I* × *A*) (Riccò 1877). Ricco’s area is defined as the area of complete spatial summation for which Ricco’s law holds true (Riccò 1877). However, in the case of larger targets, Ricco’s law does not hold; instead, only partial summation occurs, a phenomenon called Piper’s law (Howarth & Lowe 1966, Piper 1903). Piper’s law states that the threshold contrast is inversely proportional to the square root of the target area. As shown in Figure 1e, the inverse relationship is encapsulated in the data best fitted with a two-limbed model. Within Ricco’s area, the relationship between the log detection threshold and the log stimulus area exhibits a slope of −1. However, beyond this range, governed by Piper’s law, the slope becomes less than −1.

Furthermore, this inverse relationship between threshold contrast and target size in a spatial pattern is also well captured in the contrast sensitivity function (CSF), as illustrated in Figure 1c. The CSF represents a plot of contrast sensitivity as a function of spatial frequency (scale) on a log-log scale, thereby relating the visibility of a spatial pattern to both its size and contrast. The human CSF exhibits a band-pass tuning curve with a gradual roll-off at low spatial frequencies (large scale) and a steeper roll-off at high frequencies (fine scale) (De Valois et al. 1974). This characteristic shape of the CSF has been explained by the response characteristics of ganglion cell neurons (Enroth-Cugell & Robson 1966, Kelly 1975) and those of V1 cortical neurons (Carandini & Heeger 2011).

It is worth mentioning that the trade-off between threshold contrast and target size is often unheeded in typical laboratory and clinical evaluations, where one aspect (contrast or size) is fixed, while the other is measured, neglecting the interplay between the two. For instance, Pelli-Robson contrast sensitivity charts measure a person’s contrast sensitivity by determining the lowest contrast at which they can correctly read letters, while keeping the letter size constant at the recommended viewing distance. On the other hand, optotype visual acuity charts evaluate a person’s visual sensitivity, measuring the smallest letter size that they can read correctly while keeping the luminance contrast of letters constant. However, such a one-dimensional measure of visual sensitivity may not fully represent a person’s everyday visual function, particularly in real-world environments with spatial patterns varying in sizes and contrasts. This issue is particularly pertinent to individuals with compromised contrast sensitivity due to aging and/or eye diseases, as in glaucoma. For instance, it has been shown that individuals with reduced contrast sensitivity require a significantly higher text contrast to read fine print as compared to larger print, and this difference exceeds what is typically expected from those with normal contrast sensitivity (Chien et al. 2017). Similarly, individuals with reduced contrast sensitivity have been found to have a much larger Ricco’s area compared to healthy cohorts (Je et al. 2018, Redmond et al. 2010a). These findings demonstrate the interconnected nature of contrast and target size in visual processing (Figure 1c). I return to this issue in Section 4.

### 2.4. Correspondence Between the Topographic Distribution of Retinal Ganglion Cell Density and the Variation in Spatial Summation Across the Visual Field

As shown in Figure 1j, human RGC density varies across the visual field. The density reaches its peak at the fovea after adjusting for the lateral displacement of RGCs (Curcio & Allen 1990) and monotonically decreases as the distance from the fovea (i.e., eccentricity) increases. Moreover, an asymmetry in the RGC density exists between the vertical and horizontal meridians, with a significantly lower density in the inferior retina, corresponding to the upper visual field. Additionally, there is a noticeable asymmetry between the temporal and nasal retina, although this asymmetry can be obscured under binocular viewing conditions due to target projection onto both temporal and nasal retinas. Interestingly, this topographic distribution of RGC density closely mirrors the variations in human visual performance (or sensitivity) across the visual field, as illustrated in Figure 1f,g. For instance, the spatial extent to which visual information is integrated is largely determined by where the target appears in the visual field: Ricco’s area increases with eccentricity and exhibits visual-field asymmetry, with a noticeably larger area in the upper visual field compared to the rest (Kwon & Liu 2019, Volbrecht et al. 2000b, Wilson 1970).

Converging evidence suggests that, across the visual field, there appears to be an equal number of RGCs subserving the extent of the perceptive fields (i.e., a psychophysically defined summation area, such as Ricco’s area or a crowding zone) (Kwon & Liu 2019; Redmond et al. 2010b; Schefrin et al. 1998; Vasilev et al. 2003; Vassilev et al. 2003, 2005; Volbrecht et al. 2000a,b), the receptive field size of V1 cortical neurons (Fischer 1973, Kwon & Liu 2019), and a unit cortical distance in V1 (Kwon & Liu 2019, Wässle et al. 1990). These findings draw our attention to the role of the topographic distribution of RGC density in the way in which visual information is integrated in the human visual system.

By transforming the visual field into its neuronal representation in the cat’s optic tract, Fischer (1973) was the first to demonstrate that an equal number of RGCs must be stimulated to detect a visual stimulus. Fischer showed that a small spot of light on the cat’s retina (0.4 log units above the threshold of the RF midpoint) stimulates the centers of approximately 35 ganglion cells irrespective of the retinal position of the stimulus. Thus, large areas in the peripheral retina are represented by as many RGCs as small areas in the central part of the retina. On the other hand, Wässle et al. (1990) were the first to demonstrate that, to a large extent, the sampling density of RGCs in the nonhuman primate retina accounts for the logarithmic relationship between the visual field and cortical area described by the cortical magnification factor (i.e., the amount of cortical area dedicated to a given visual field area or millimeters of cortical surface per degree of visual angle).

Such close correspondence between the topographic distribution of RGC density and the perceptive fields or cortical magnification factor has also been demonstrated in human studies (Kwon & Liu 2019; Redmond et al. 2010b; Rovamo 1978; Vassilev et al. 2005; Volbrecht et al. 2000a,b; Watson 1987). A recent study by Kwon & Liu (2019) lends support to the idea that RGC density in the human retina contributes to the nonuniform cortical representation of the visual field. They determined the quantity of RGCs subserving Ricco’s area (or the crowding zone) across the visual field by multiplying the measured Ricco’s area (or crowding zone) with the corresponding RGC density (Drasdo et al. 2007) (Figure 1j), as derived from previous histological studies of the healthy adult human retina (Curcio & Allen 1990). Their results showed an excellent correspondence between the sampling density of RGCs and the variation in Ricco’s area (89–95% explained variance) and the crowding zone (81–97% explained variance). In other words, the number of RGCs that underlie Ricco’s area (or the crowding zone) remains relatively constant, with approximately 14 RGCs for Ricco’s area and 6,700 RGCs for the crowding zone, across the visual field (Figure 1k). This suggests that, regardless of the target location in the visual field, an equal number of RGCs appears to be responsible for the critical area of spatial summation, provided that other factors, such as background luminance or stimulus duration, are held constant. Kwon & Liu further demonstrated that, when accounting for the topographic inhomogeneity of the RGC density across the retina, it becomes possible to express both the V1 cortical magnification factor and the eccentricity-dependent increase in the RF size of V1 cortical neurons in terms of a constant number of RGCs. For example, the number of midget RGCs corresponding to the size of a neuron’s classical RF in nonhuman primates (Gattass et al. 1981) or the functional magnetic resonance imaging population RF in human V1 (Dumoulin & Wandell 2008) are estimated to be approximately 29, and these estimates remain consistent regardless of eccentricity (Figure 1l). Similarly, the number of midget RGCs underlying a 1-mm cortical distance remains consistent at approximately 12 (Figure 1m). Kwon & Liu further substantiated their findings by employing a retina-V1 pooling model based on statistical sampling of the human RGC mosaic and probabilistic connections between cortical layers. Consistent with their empirical results, their simulations also showed that each V1 simple cell neuron, i.e., the output of the model, receives the same fixed number of inputs from RGCs regardless of eccentricity.

Furthermore, a number of human psychophysical studies (Redmond et al. 2010b; Schefrin et al. 1998; Vasilev et al. 2003; Vassilev et al. 2003, 2005; Volbrecht et al. 2000a,b) have also provided compelling evidence that an equal number of RGCs is involved in Ricco’s area across the visual field. For instance, Volbrecht et al. (2000a) measured Ricco’s area across a range of retinal eccentricities from 0° to 20° for S- and L-cone mechanisms to determine whether the size of Ricco’s area could be explained by either photoreceptor or RGC density. They found that, as retinal eccentricity increases from 8° to 20°, the size of Ricco’s area associated with each cone mechanism grows. This pattern provides strong support for the role of RGC density, rather than cone density, in determining the size of Ricco’s area, as there is little change in cone density beyond the range of 8–10° eccentricity. On the other hand, using isoluminant S-cone chromatic vision, Vassilev et al. (2005) also showed that a fixed number of small bistratified ganglion cells (3 to 4) underpins Ricco’s area measured at eccentricities from 5° to 30°.

It is worth noting that the size of Ricco’s area can be influenced by other factors, such as background luminance (Barlow 1958, Redmond et al. 2013), wavelength (King-Smith & Carden 1976, Volbrecht et al. 2000a), or stimulus duration (Mulholland et al. 2015, Wilson 1970). As a result, the specific number of RGCs subserving Ricco’s area may vary under different experimental conditions. Nevertheless, the number of RGCs underlying Ricco’s area likely remains unchanged across the visual field when other factors are held constant (as indicated by the change in the intercept, rather than the slope, in Figure 1k). These findings collectively illuminate a quantitative agreement between the topographic distribution of the RGC density and the nonuniform spatial integration across the visual field. They further highlight the significant computational constraints set by the RGCs during the early encoding stage of visual input. These findings thus raise the intriguing possibility that the fixed number of RGCs rule may serve as one of the organizing principles of the human visual system (Figure 1f).

## 3. GANGLION CELL DAMAGE IN THE MACULA

### 3.1. Measurement and Quantification of Ganglion Cell Loss

The loss or dysfunction of RGCs in the macular region can lead to central vision deficits, significantly impacting a person’s quality of life. Thus, accurately quantifying macular RGC damage is critical not only for monitoring disease progression, but also for a more comprehensive assessment of patients’ daily visual dysfunction.

**Figure 2:**
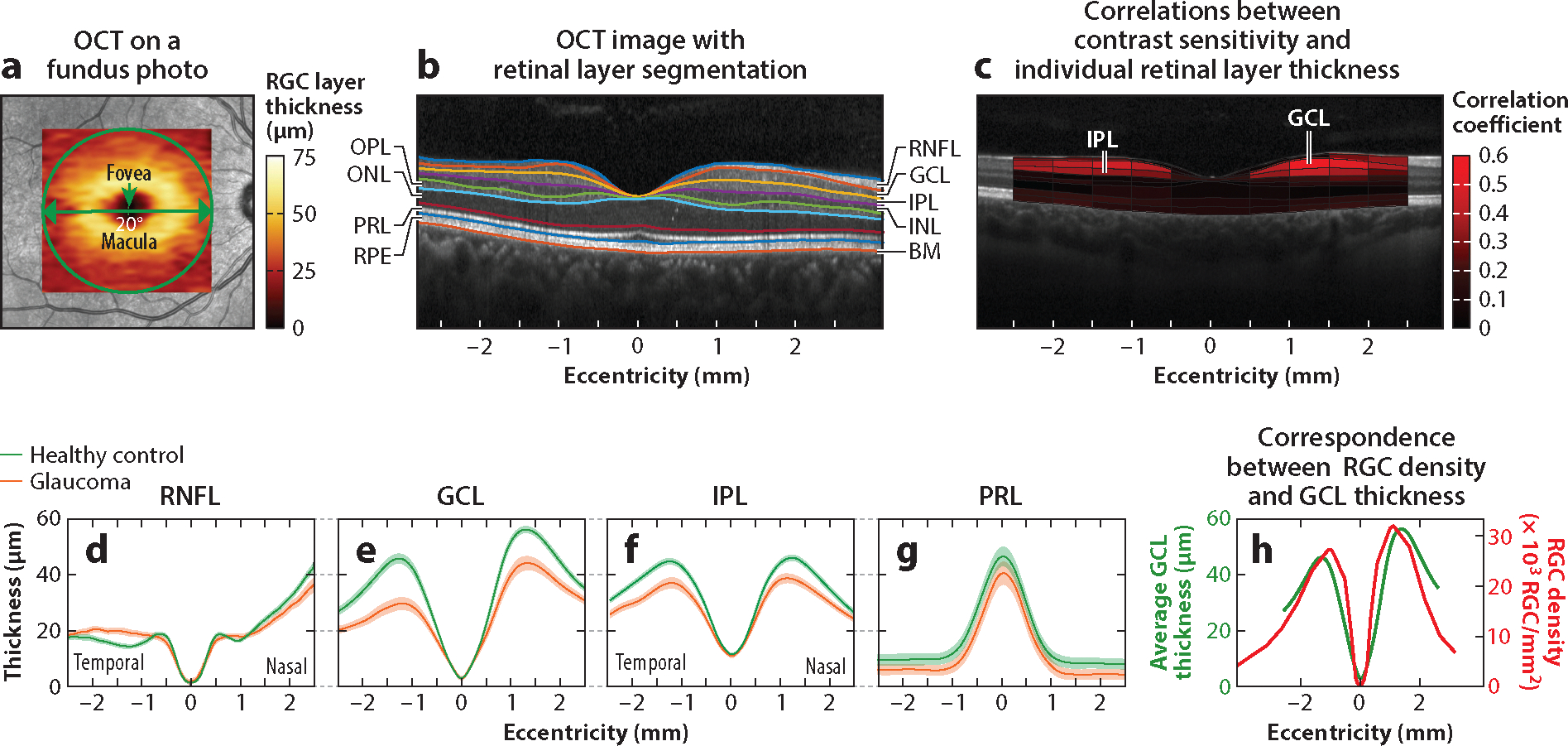
Glaucomatous ganglion cell damage in the macula. (*a*) An example of macular spectral domain–optical coherence tomography (SD-OCT) scans showing an overlay of the ganglion cell layer (GCL) thickness map on a fundus photograph. (*b*) An example of an SD-OCT B-scan cross-sectional image with retinal layer segmentation: the retinal nerve fiber layer (RNFL), containing the axons of ganglion cells; the GCL, containing the cell bodies of ganglion cells and displaced amacrine cells; the inner plexiform layer (IPL), containing synaptic connections between the dendrites of ganglion cells and the axons of bipolar cells; the inner nuclear layer (INL), containing the cell bodies of bipolar, horizontal, and amacrine cells; the outer plexiform layer (OPL), containing synaptic connections between horizontal and photoreceptor cells; the outer nuclear layer (ONL), containing the cell bodies of the rods and cones; the photoreceptor layer (PRL), containing the inner and outer segments of photoreceptors; and the retinal pigment epithelium (RPE) layer, comprising pigment cells, situated above Bruch’s membrane (BM). (*c*) Correlations between foveal contrast sensitivity and individual retinal layer thickness. (*d*–*g*) The thickness of individual retinal layers compared between glaucoma (*orange*) and age-matched healthy vision (*green*). (*h*) The correspondence between the retinal ganglion cell (RGC) density (without adjusting for RGC displacement) and the thickness of the GCL across the retina. The average thickness of the GCL (*green line* on the left *y* axis) is juxtaposed with the RGC density (*red line* on the right *y* axis) from a histological study of the adult human retina (Curcio & Allen 1990). Data sourced and figure adapted from Shamsi et al. (2022b) (CC BY-NC-ND 4.0).

Early studies utilized nonhuman primate models of experimental glaucoma or postmortem human retinas from glaucoma patients to quantify the relationship between visual sensitivities and RGC loss. The RGC loss was typically determined through histological counts of corresponding retinal sections or by assessing remaining axons in the optic nerve (Chauhan et al. 2002, Fileta et al. 2008, Harwerth et al. 1999). On the other hand, recent advances in adaptive optics imaging techniques have unveiled the great potential for morphometric analysis of ganglion cell layer (GCL) somas in the living human retina (Liu et al. 2017b). Nevertheless, the direct counting of RGCs in the living human eye remains technically challenging, with no commercially available off-the-shelf products for this purpose. However, RGC loss can be inferred by measuring the thickness of the RGC layer using OCT, a noninvasive imaging method that utilizes low-coherence interferometry to generate in vivo, cross-sectional images of ocular tissues with a high axial resolution (Huang et al. 1991) (Figure 2a,b).

The human retina consists of several layers of retinal neurons interconnected by synapses and is supported by an outer layer of pigment epithelial cells. As shown in Figure 2b, this laminar organization of the retina can be observed with OCT. As the structural characteristics of the retina, such as the thickness and reflectivity of each layer, often indicate dysfunction and degeneration of retinal neurons, technical advancements in OCT devices revolutionized the way in which glaucomatous damage is detected and assessed (Huang et al. 1991). In particular, recent OCT devices come equipped with software that enables automatic segmentation and thickness measurements of intraretinal layers in the macular region (Tan et al. 2008), allowing for more precise and objective measurements of macular structures. In Figure 2b, you can see an OCT cross-sectional image of the retina, detailing the three innermost retinal layers preferentially affected by glaucoma: the retinal nerve fiber layer (RNFL), GCL, and inner plexiform layer (IPL). These layers contain the axons, cell bodies, and dendrites, respectively, of the ganglion cells.

Recent macular OCT imaging studies have shown a correlation between the thicknesses of the macular GCL and IPL (GCL+IPL) and either RGC counts in the macula or perimetric sensitivity in the central 10° of the visual field. For example, Zhang et al. (2014) investigated the structural and functional relationship in the macular region using data from 77 healthy eyes, 154 eyes with glaucoma suspect, and 159 glaucomatous eyes. They estimated macular RGC counts using an empirical model that leveraged perimetric sensitivity and OCT of the circumpapillary RNFL, revealing a 41% reduction in estimated macular RGCs in glaucomatous eyes compared to healthy eyes. They also observed a strong correlation between estimated macular RGC counts and macular GCL+IPL thickness (*r*^*2*^ = 0.65, *p* < 0.001).

Furthermore, Raza & Hood (2015) investigated the macular structure and function relationship in glaucomatous eyes, using RGC counts estimated from macular RGC layer thickness in combination with published histological RGC density (counts/mm^2^). They found that macular RGC estimates, indicated by RGC layer thinning, were in good agreement (Spearman’s *p* = 0.26–0.47) with sensitivity loss observed in a 2-degree grid (10–2 perimetry test).Greenfield et al. (2003) also reported a significant correlation (*r*^*2*^ = 0.47; *p* < 0.001) between macular thickness derived from OCT and visual field defects [mean deviation (MD) values from perimetry] in glaucomatous eyes. As demonstrated in Figure 2h, these findings collectively support the use of macular RGC layer thinning as a surrogate measure for RGC loss or degeneration in the macula.

### 3.2. Overlooked Macular Damage

As glaucoma is commonly associated with ONH damage, primary detection and management methods focus on measuring intraocular pressure and assessing structural changes in the ONH, typically located approximately 15–17° away from the fovea on the nasal retina. For instance, OCT-based glaucoma testing predominantly relies on optic disk scans to analyze the cupping of the ONH and assess the thickness and volume of the RNFL surrounding the ONH. Although reducing intraocular pressure is crucial for managing glaucoma and preventing further optic nerve damage, this treatment focus might inadvertently lead to inadequate attention to changes in the macula, thus overlooking these significant factors (Wang et al. 2015).

Furthermore, clinical perimetry commonly employs testing protocols that prioritize the detection of peripheral visual field defects, such as the HFA 24–2 or 30–2 tests, rather than those focusing on central visual defects, like the HFA 10–2 test. The 24–2 and 30–2 protocols, featuring test points spaced 6° apart, include only four test points in the central 10° visual field, resulting in an undersampling of this crucial area. Moreover, the use of a standard target stimulus in perimetry (e.g., a 0.43° diameter) may not be optimal for detecting visual defects in the central region. This inadequacy arises because the critical area of spatial summation (i.e., Ricco’s area) in the foveal region is notably smaller (approximately 0.1° in diameter) compared to the typical target size (Inui et al. 1981). Consequently, such clinical practices could lead to an overestimation of peripheral vision loss while underestimating central vision loss. In addition, patient and public awareness campaigns emphasizing peripheral vision loss in glaucoma might further contribute to this oversight.

### 3.3. Macular Damage Unveiled Through Recent Optical Coherence Tomography Studies

Over the past decade, the interest in macula damage has surged, partly due to significant advancements in OCT technology (Hood et al. 2013). Hood and his colleagues have played a leading role in shedding light on the presence of macular damage across various stages of glaucoma and in characterizing this damage (for a review of OCT studies on macula damage in glaucoma, see also Hood et al. 2013). Several OCT imaging studies utilizing macular scans have pinpointed a significant loss of RGCs in the macular region of glaucomatous eyes (de A Moura et al. 2012; Glovinsky et al. 1993; Hood et al. 2012, 2013, 2014) as compared to age-matched healthy eyes. This loss is indicated by the thinning of the GCL and IPL, where ganglion cell bodies and their dendritic structures are situated.

For instance, Wang et al. (2009), using frequency domain–OCT, showed that, in glaucoma patients, both the RNFL and the RGC plus IPL (RGC+) in the macula were significantly thinner as compared to healthy controls. Similarly, Chien et al. (2017), using spectral domain (SD)-OCT, observed a 20% decrease in the thickness of the RGC+ layer in the macular region of early and moderate glaucomatous eyes compared to age-matched healthy eyes, even after accounting for factors like pupil diameter and visual acuity. In agreement with these studies, Shamsi et al. (2022b) observed similar findings by utilizing macular SD-OCT scans to acquire the thickness profile of individual retinal layers across eccentricities (Figure 2d–g). Their results revealed significant thinning of layers associated with RGCs, particularly the GCL and IPL (*p* < 0.01), in glaucomatous eyes compared to age-matched healthy eyes. In contrast, no significant differences were observed in the other layers, such as the photoreceptor layer, inner nuclear layer, outer plexiform layer, and outer nuclear layer, when compared to those in age-matched healthy eyes.

Importantly, mounting evidence suggests that glaucomatous damage involves the macular region, even in the early stages of the disease. For example, Hood et al. (2012) conducted a study that compared the thickness profiles of the RGC+ layer and the RNFL in the eyes of glaucoma suspects or glaucoma patients to those in healthy eyes. Their findings showed that the average RGC+ layer is significantly thinner even in patients without apparent visual field defects in standard perimetry. Moreover, this layer progressively becomes thinner with decreasing perimetric sensitivity. The thinning of the GCL mostly occurred within the central four points of the 24–2 test (corresponding to the central 6° of the visual field), even after compensating for the RGC displacement. Similar findings were also reported in other studies (Takagi et al. 2012, Tan et al. 2009) demonstrating thinning of the macular ganglion cell complex, which includes the RNFL, GCL, and IPL, in individuals with a visual field classified as normal. Collectively, these findings show that early macular RGC damage is more common than was previously thought (Hood et al. 2011), with studies reporting its presence in up to 80% of patients with mild glaucoma (Blumberg et al. 2019, Hood et al. 2014).

## 4. FUNCTIONAL CONSEQUENCES OF MACULAR GANGLION CELL DAMAGE FOR CENTRAL VISUAL FUNCTION

Consistent with the structural damage, both subjective reports and objective functional measurements have provided additional support for deficits in central visual function in glaucoma patients. These deficits include impaired foveal contrast sensitivity (Bambo et al. 2016, Chien et al. 2017, Hawkins et al. 2003, Horn et al. 1995, Ichhpujani et al. 2020, Lahav et al. 2011, Wilensky & Hawkins 2001), increased visual crowding (Ogata et al. 2019; Shamsi et al. 2021, 2022a; Stievenard et al. 2021), decreased visual span (Kwon et al. 2017), reduced functional field of view (Shamsi et al. 2021), reading difficulty (Burton et al. 2012; Goddin et al. 2023; Ikeda et al. 2021; Kwon et al. 2017; Mathews et al. 2015; Ramulu et al. 2009, 2013), and impaired object and face recognition (Glen et al. 2012, Hirji et al. 2021, Issashar Leibovitzh et al. 2023, Lenoble et al. 2016, Roux-Sibilon et al. 2018). In particular, these deficits appear to be more pronounced under conditions of higher sensory and cognitive demands, such as low luminance or divided attention (Bhorade et al. 2013, Blumberg et al. 2019, Shamsi et al. 2021, Stievenard et al. 2021).

**Figure 3:**
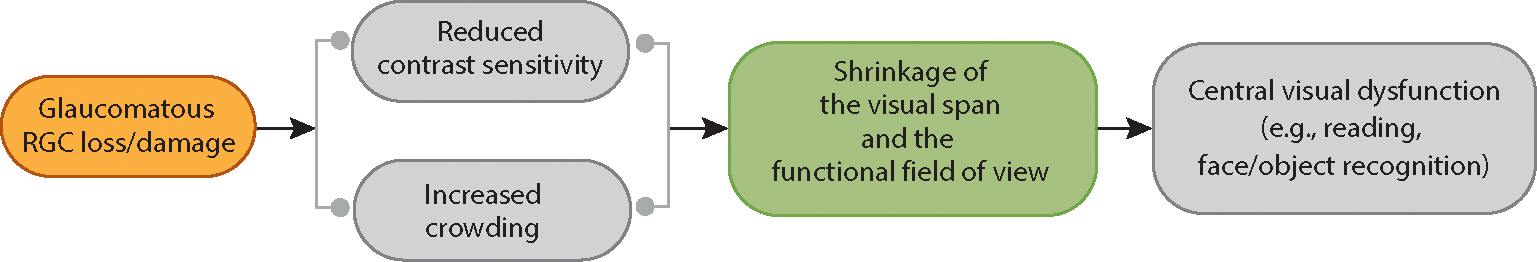
Functional consequences of macular ganglion cell damage. The flow chart illustrates potential perceptual mechanisms elucidating how glaucomatous retinal ganglion cell (RGC) damage may impact tasks reliant on central vision, such as reading or object and face recognition.

In this section, I discuss, as depicted in Figure 3, how glaucomatous RGC damage affects low-level visual function, including contrast sensitivity, crowding, and the visual span, which together may contribute to difficulties in tasks requiring central vision, such as reading or recognizing objects and faces.

### 4.1. Reduced Contrast Sensitivity in Central Vision

Considering the pivotal role of RGCs in contrast coding, it is unsurprising that a substantial deficit in contrast sensitivity has been found in glaucoma patients (Bambo et al. 2016, Chien et al. 2017, Hawkins et al. 2003, Horn et al. 1995, Ichhpujani et al. 2020, Klein et al. 2015, Lahav et al. 2011, Liu & Kwon 2020, Xiong et al. 2020, Wilensky & Hawkins 2001). Contrast sensitivity, the ability to detect differences in contrast, is a key building block of human pattern vision and, thus, crucial to various visual activities (Owsley 2003). Contrast sensitivity is commonly assessed using optotypes or gratings with varying luminance contrasts, often measured with tests like the Pelli-Robson letter charts, the Mars letter charts, the CSV-1000, or the VCTS contrast sensitivity charts. As detailed in Section 2.2, the CSF refers to the measurement of contrast sensitivity across spatial frequencies. As depicted in Figure 1c, the human CSF is characterized by a band-pass tuning curve with a gradual roll-off at low spatial frequencies and a steeper roll-off at high frequencies (De Valois et al. 1974).

A study by Lin et al. (2018) underscored the importance of assessing foveal contrast sensitivity in glaucoma. They demonstrated that, among various vision measures, such as visual acuity, color vision, and stereoacuity, foveal contrast sensitivity emerged as an indispensable factor in evaluating visual disability in glaucoma patients. Indeed, many studies have reported significant foveal contrast sensitivity deficits, even in early or moderate stages of glaucoma (Bambo et al. 2016, Chien et al. 2017, Hawkins et al. 2003, Horn et al. 1995, Ichhpujani et al. 2020, Klein et al. 2015, Lahav et al. 2011, Liu & Kwon 2020, Sohail et al. 2023, Xiong et al. 2020). For instance, Lahav et al. (2011) found that contrast sensitivity was significantly reduced in glaucomatous eyes under both photopic (daylight) and mesopic (dim light) conditions. On the other hand, other studies showed that reduced contrast sensitivity in glaucoma becomes more apparent in mesopic or low-luminance conditions (Bierings et al. 2019, Hertenstein et al. 2016). It has been shown that suppressive or inhibitory surround mechanisms become weaker under dim or mesopic light conditions (Barlow 1958, Barlow et al. 1957, Cowan et al. 2017), which may result in compromised contrast coding under low-luminance conditions.

Importantly, contrast sensitivity deficits can occur despite seemingly normal visual acuity. Wilensky & Hawkins (2001), for instance, demonstrated that eyes with glaucoma, presenting a visual acuity of 20/40 or better, exhibited reduced contrast sensitivity, which was significantly associated with increased visual field defects (MD) (*r* = 0.638, *p* < 0.05). They further speculated that the impaired contrast sensitivity in glaucoma patients may explain their reported vision problems despite having normal or near-normal visual acuity. Furthermore, Chien et al. (2017) showed that, in comparison to healthy cohorts, individuals with mild or moderate glaucoma required significantly higher threshold contrasts to reliably recognize letters at various eccentricities (0° by 70.2%, *p* = 0.011; 3° by 69.5%, *p* < 0.001; 6° by 70.9%, *p* < 0.001) when adjusting the letter size based on the cortical magnification factor for each test location.

Although most studies on contrast sensitivity in glaucoma have used broadband letter charts or focused on a single spatial frequency, some have examined the full CSF of glaucomatous vision (El-Gohary & Siam 2009, Klein et al. 2015, Kwon & Liu 2021, McKendrick et al. 2007). For instance, using the VCTS 6500 chart (i.e., a grating chart that measures contrast sensitivity at a wide range of spatial frequencies), El-Gohary & Siam (2009) evaluated binocular contrast sensitivity across five spatial frequencies [1.5, 3, 6, 12, and 18 cycles per degree (cpd)] in early glaucoma patients and healthy controls. Their findings showed a significant reduction in the CSF of glaucomatous vision across all spatial frequencies compared to healthy controls.

Similarly, Kwon & Liu (2021) investigated the CSF across eight spatial frequencies ranging from 0.5 to 18 cpd and found significant contrast deficits in glaucoma with no interactions between glaucoma and spatial frequency. Klein et al. (2015) also observed reduced contrast sensitivity in early glaucoma at spatial frequencies of 0.5, 1.5, and 3 cpd when compared to healthy controls, with a consistent 0.2 log unit difference regardless of spatial frequency, even after adjusting for cataract type and severity. In contrast, McKendrick et al. (2007) reported significant contrast deficits only at affected midperipheral locations, observing no deficits at the fovea for spatial frequencies of 0.25–2 cpd. The apparent discrepancies in the findings regarding the interaction between spatial frequency and contrast sensitivity in glaucoma warrant future studies under more standardized experimental conditions.

On the other hand, Shamsi et al. (2022b) utilized a deep learning approach to explore the relationship between an individual’s foveal contrast sensitivity and specific retinal layers housing RGCs. Their study involved acquiring macular OCT scans and Pelli-Robson contrast sensitivity data from 225 subjects, including those with glaucoma, age-related macular degeneration, or age-matched normal vision. They trained a deep convolutional neural network to predict a person’s contrast sensitivity solely based on their retinal imaging data measured via OCT scans (Figure 2b). By computing the network’s gradient-weighted regression activation map, which represents the features the network relies on most for the output prediction, they identified that the retinal layers containing RGCs were the critical features used by the network to predict contrast sensitivity. Moreover, they observed a significant correlation between the thickness of the GCL and the IPL and contrast sensitivity in the macular region (Figure 2c). These findings further confirm the significant role of RGCs in determining human contrast sensitivity while illuminating significant contrast deficits in the central vision of individuals with glaucoma.

### 4.2. Increased Crowding in the Central Visual Field

As discussed in Section 2.4, a fixed number of RGCs has been shown to subserve the critical area of spatial summation across the visual field, such as Ricco’s area and crowding zone, suggesting a close linkage between RGC density and the extent of spatial summation. It has been proposed that, in the presence of glaucomatous ganglion cell loss, the visual system might compensate for this loss by integrating signals over a larger area to maintain threshold detectability (Pan & Swanson 2006). This view aligns with findings from psychophysical studies that have demonstrated an inverse relationship between threshold stimulus size and RGC density (Chauhan et al. 1999, Fellman et al. 1989, Wall et al. 1991). According to this proposition, glaucomatous RGC loss likely brings about an enlargement in the critical area of spatial summation. Empirical studies, indeed, have supported this view, showing alterations in spatial summation mechanisms following glaucomatous damage. For instance, Redmond et al. (2010a) reported a significant enlargement of Ricco’s area in early glaucoma compared to healthy normal vision. Mulholland et al. (2015) observed a similar increase in Ricco’s area. Moreover, King et al. (2006) offered insights into the neural basis of changes in spatial summation following glaucomatous damage. Their study also revealed that experimentally induced ganglion cell death led to an enlargement in the size of cortical RFs in the adult rat brain. This observed increase in RFs was directly proportional to the degree of glaucomatous damage, further highlighting the close linkage between the extent of spatial summation and ganglion cell damage or loss.

**Figure 4:**
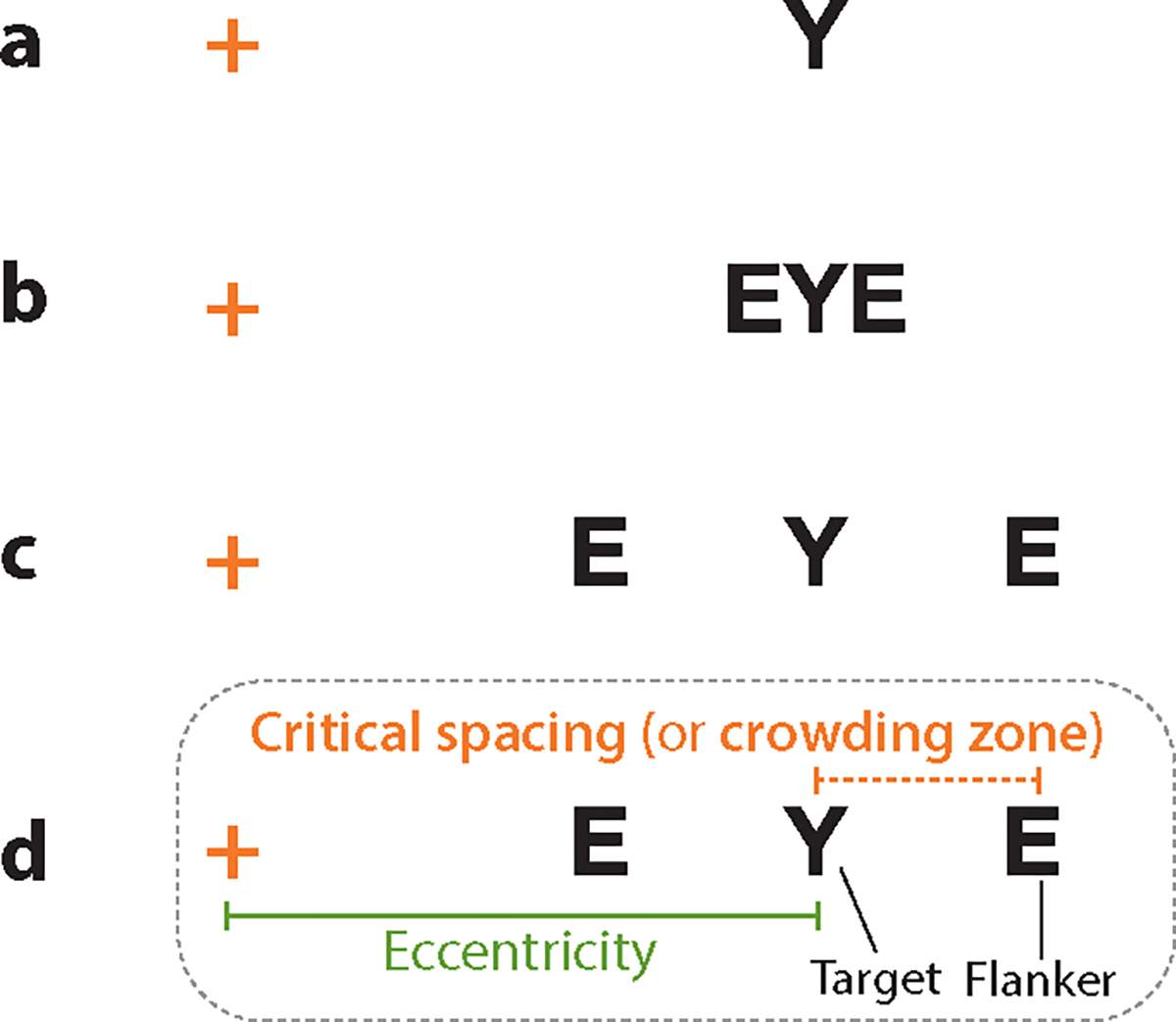
Visual crowding. (*a*) While fixating on the orange cross, a person can easily identify the isolated letter on the right, (*b*) but may find it impossible to identify the middle letter appearing at the same retinal eccentricity. (*c*) However, crowding can be alleviated by increasing the spacing between the target and flankers. (*d*) The effect of crowding is often assessed by measuring critical spacing, also known as crowding zone, i.e., the minimum center-to-center spacing between the target and flankers that allows for reliable target recognition.

An enlargement in the critical area of spatial integration in the presence of glaucomatous damage has also been found in a perceptual phenomenon called visual crowding. Visual crowding refers to the inability to recognize an individual target when it is surrounded by clutter or nearby items. Visual crowding is common in natural scenes and impacts a wide range of everyday visual tasks (Bouma 1970, Pelli & Tillman 2008). The effect of crowding is demonstrated in Figure 4. While fixating on the orange cross, a person can easily identify the isolated letter on the right (Figure 4a) but may find it impossible to identify the middle letter (Figure 4b) at the same retinal eccentricity. This is due to the interference from the nearby letters disrupting the recognition of the target. Since the same target is recognizable when presented alone, visual crowding cannot simply be explained by reduced visual acuity or contrast sensitivity at a given retinal location. It is often attributed to the erroneous integration of visual features due to either an excessively large integration zone at a preattentive level (Pelli et al. 2004) or a coarse spatial resolution of attention (He et al. 1996).

The magnitude of crowding can be assessed by comparing the difference in recognition accuracy between crowded and uncrowded (single) target conditions (i.e., decreased accuracy for crowded conditions). Alternatively, one can directly measure the critical spacing or crowding zone, which is the minimum spacing between a target and flankers required for reliable target identification, as shown in Figure 4d. In this case, greater crowding corresponds to a larger crowding zone. Similar to Ricco’s area, crowding zone also increases with increasing eccentricity. Crowding is known to be more pronounced in peripheral vision, while little crowding exists in foveal vision (Toet & Levi 1992). However, certain visual disorders, such as amblyopia, exhibit significantly greater foveal crowding compared to what is observed in normal vision (Bonneh et al. 2007, Hariharan et al. 2005).

Accumulating evidence suggests that glaucomatous damage exacerbates visual crowding (Ogata et al. 2019, Shamsi et al. 2022a, Stievenard et al. 2021, Stringham et al. 2020). Ogata et al. (2019) measured the crowding zone at 10° eccentricity in glaucoma patients and healthy controls. Their results indicated a significant increase in crowding in glaucoma patients, even those with only mild visual field defects on standard perimetry. The observed crowding zone exhibited an approximately 17% increase in glaucoma patients compared to the healthy control group. In addition, the magnitude of crowding was significantly correlated with the amount of neural loss quantified by OCT. Furthermore, Shamsi et al. (2022a) observed a 21% increase in crowding zone in the parafoveal and perifoveal vision (i.e., 2° and 4° retinal eccentricities) of patients with early or moderate glaucoma compared to that of age-matched healthy controls (Shamsi et al. 2022a). Importantly, the comparison of the crowding zone between the worse and the better eye of glaucoma patients further revealed that eyes with more severe glaucomatous damage, as determined by HFA 10–2 perimetry, exhibited a significantly larger crowding zone. However, in early or moderate glaucomatous eyes, there appears to be limited evidence supporting increased crowding in the foveal region at less than 0.25° eccentricity. Shamsi et al. (2022a) measured foveal crowding zone in patients with early or moderate glaucoma and age-matched healthy controls using Pelli’s foveal crowding paradigm (Pelli et al. 2016). They found that the average crowding zone was 0.061° for glaucomatous vision and 0.056° for age-matched normal vision at the fovea, but the difference between the two groups was not statistically significant. On the other hand, Stievenard et al. (2021) reported increased foveal crowding in glaucoma compared to age-matched normal vision. In their study, participants were tasked with determining whether a mouth presented within a face (crowded) or in isolation (uncrowded) was open or closed. Unlike the control group, which generally exhibited higher accuracy when the mouth was presented in a face (i.e., displaying the face superiority effect), 10 of 17 glaucoma patients performed more accurately in the isolated mouth condition (uncrowded condition) for small images of less than 1° visual angle. Considering these empirical findings, increased crowding appears to be evident in the central visual field of glaucomatous vision, even in its early and moderate stages.

### 4.3. Shrinkage of the Functional Field of View and the Visual Span

The human visual field is a region of space that encompasses approximately 200° horizontally and approximately 130° vertically (Wolter 1972). It is composed of distinct zones—the foveal, parafoveal, perifoveal, and peripheral regions—each serving a specific function. Foveal vision offers fine, detailed information, while the surrounding regions—parafoveal, perifoveal, and peripheral vision—provide coarser but broader contextual information that is crucial for guiding eye movements and subsequent visual processing. As a result, these regions play a significant role in various everyday activities, including reading (McConkie & Rayner 1975), visual search (Wolfe et al. 2017), scene recognition (Larson & Loschky 2009), maintaining postural balance (Zwierko et al. 2020), and driving (Ball et al. 1993, Huisingh et al. 2015). However, the extent of an area visible to an individual depends on the sensory and cognitive demands of given tasks, contrary to our perception of an extensive visible world. In this section, I discuss how glaucomatous RGC damage increases susceptibility to such sensory and cognitive demands, resulting in a reduced field of view.

Glaucomatous visual field loss is typically evaluated using standard perimetry, assessing an individual’s ability to detect a small spot of light against a uniform white background across the visual field. This method, however, may not fully capture the patient’s ability to process visual information in a dynamic visual environment (Crabb 2016). Day-to-day tasks frequently involve various visual challenges, such as clutter (crowding), managing multiple tasks simultaneously (divided attention), and responding within time constraints (time-sensitive responses). In particular, the ability to split or divide attention has been closely associated with real-life activities—such as attending to peripheral objects while fixating on central visual inputs (Owsley & McGwin 1999, Tatham et al. 2014). The detrimental effects of divided attention are more pronounced in older adults or individuals with visual impairments (Lee et al. 2020, Tatham et al. 2014). For example, Tatham et al. (2014) demonstrated a correlation between glaucomatous structural damage and decreased ability to divide attention in simulated driving conditions. This led to significantly slower reaction times in glaucoma patients compared to age-matched healthy controls. The Useful Field of View (UFOV) test, developed by Ball and colleagues (Ball & Owsley 1993, Ball et al. 1988, Sekuler & Ball 1986), evaluates an individual’s visual performance in discriminating target stimuli amid divided attention or visual distractors by assessing their duration thresholds, i.e., the minimum time required to complete a task with a criterion accuracy of 75% (Owsley 2013). The UFOV test has been identified as a strong predictor of driving ability and crash risk (Sekuler & Ball 1986, Shakarchi et al. 2019, Wood & Owsley 2014). Studies have shown that individuals with even mild or moderate glaucoma exhibited reduced visual search and identification performance in the UFOV test compared to healthy control groups (Haymes et al. 2007, Lee et al. 2020).

**Figure 5:**
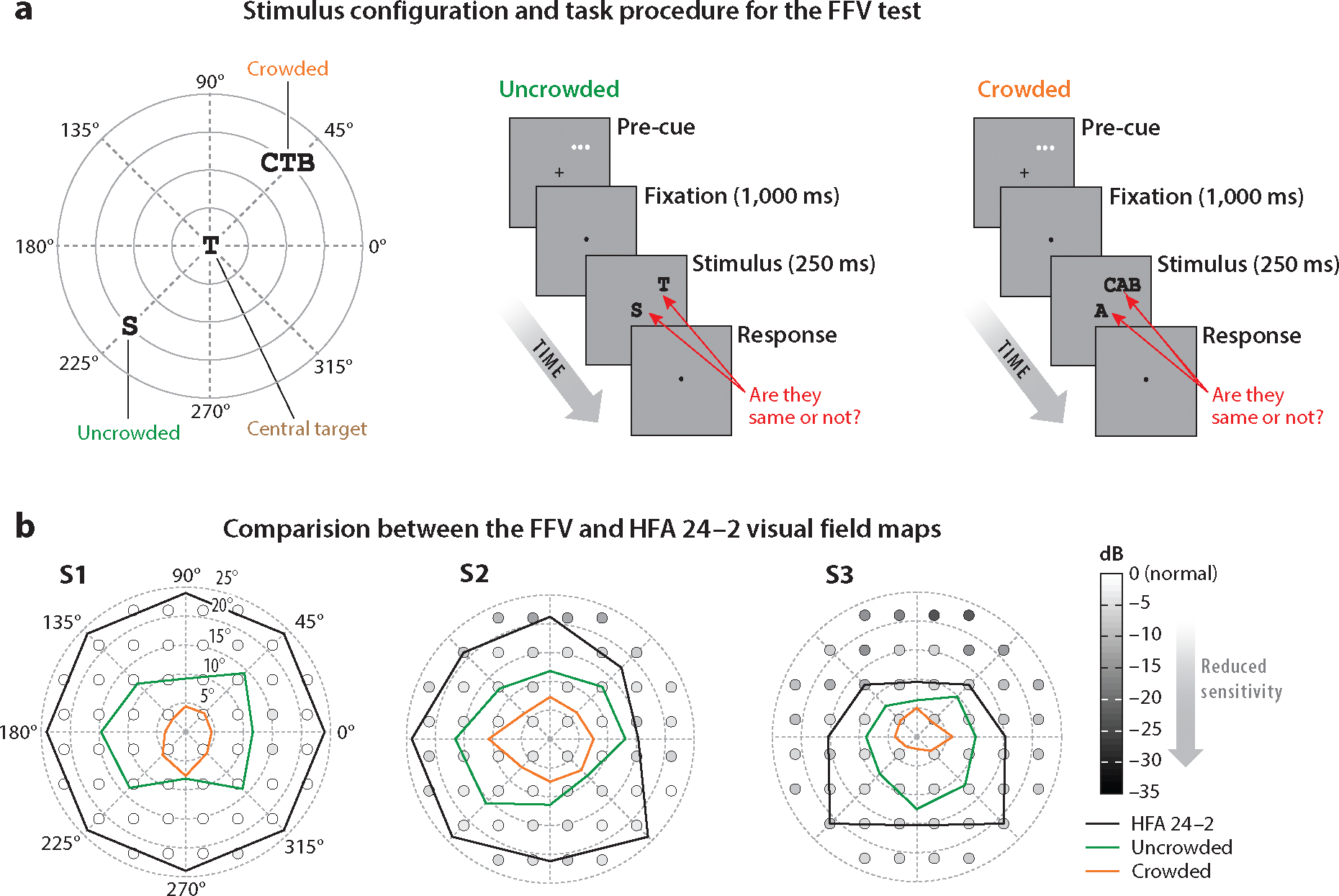
The FFV test. (*a*) Stimulus configuration and task procedure of the FFV test. (*b*) Comparison between the outcomes of the FFV and those of standard perimetry (i.e., the HFA 24–2 test, *black line*). On the polar coordinate plane, the spatial extent of the FFV under both the crowded (*orange line*) and uncrowded (*green line*) conditions is depicted. The spatial extent determined by the HFA 24–2 test is also shown with a black solid line, delineating the visible boundaries established based on a criterion of spot-light sensitivity. Data sourced and figure adapted from Shamsi et al. (2021) (CC BY-NC-ND 4.0). Abbreviations: dB, decibel; FFV, Functional Field of View; HFA, Humphrey Field Analyzer.

While the UFOV test utilizes visual processing speed as an outcome metric, the Functional Field of View (FFV) test, developed by Shamsi et al. (2021), estimates visible boundaries or area across the visual field. These boundaries, as depicted in Figure 5, delineate the maximal extent of the central visual field where an individual can reliably identify a peripheral target under cognitive or sensory demands. In the FFV test, observers are presented with a peripheral target either in isolation or among nearby distractors (Figure 5a). They are instructed to determine whether the single letter (uncrowded) or the middle letter in letter triplets (crowded) matches the concurrently displayed letter at the central fixation region. This task, therefore, necessitates attention to both locations (divided attention). Through a series of adaptive trials, a plot of the proportion of correct responses (e.g., 79% accuracy) against the target location is constructed, resulting in a visual-field map (Figure 5b). As shown in Figure 5b, Shamsi et al. (2021) found that both glaucoma patients and age-matched normal cohorts have greater difficulty in recognizing a target in clutter beyond the central 10° visual field under divided attention. In contrast, when the target was presented in isolation, recognition extended to the central 20° visual field. Their findings, therefore, demonstrated that visual crowding (clutter) has the potential to reduce the functional field of view by an average of 50%, underscoring the constraints imposed by crowding on the extent of the visual field available for target recognition. The extent of this reduction was notably greater among glaucoma patients when compared to both healthy young adults and older adults, suggesting a greater susceptibility in glaucoma patients to tasks involving visual clutter and divided attention under constrained time limits. Importantly, as depicted in Figure 5b, the field of view predicted by standard perimetry was noticeably larger than that obtained from the FFV test. This discrepancy underscores the importance of assessing the field of view in a manner that represents real-world tasks.

Furthermore, it has been shown that glaucomatous damage reduces the size of the visual span, which refers to the number of letters that are reliably recognizable in a single glance (Kwon et al. 2017). The visual span can be thought of as a window within the visual field where letters can be reliably recognized (Legge et al. 2001). The visual span is commonly measured through a letter-recognition task involving trigrams—sets of three random letters briefly flashed (e.g., 200 ms) at varying positions to the left and right of the fixation while the subject maintains central fixation. For healthy adults with normal vision, the visual span is known to be approximately 9–14 letters (Legge et al. 1997). As the visual span is largely limited by visual crowding, it is often termed the uncrowded window for object recognition (Schotter et al. 2012). Several studies have demonstrated a close linkage between reading speed and visual span size in both healthy individuals and clinical populations (Cheong et al. 2008, Kwon et al. 2007, Legge et al. 1997, Liu et al. 2017a). Kwon et al. (2017) investigated the impact of glaucomatous damage on the visual span. Their findings demonstrated that individuals with mild or moderate glaucoma exhibited a significantly reduced visual span (by 11 bits, equivalent to more than two characters at each fixation) compared to age-matched healthy controls, despite having normal binocular visual acuity (20/20 Snellen equivalent). Moreover, this reduction in the visual span was closely related to slower reading speed in glaucoma patients (*r* = 0.7, *p* < 0.01), accounting for 50% of the variance in reading speed.

Together, these findings underscore the importance of evaluating the field of view under varying attentional demands or amid distractors, as these conditions better represent the sensory and cognitive demands encountered in daily activities.

### 4.4. Reading Difficulty

Reading is indispensable to our everyday lives and integral to a variety of activities—including reading books and newspapers, interpreting traffic and street signs, navigating interfaces on mobile or desktop devices, and reading menus or food labels. As such, reading is a key component of our overall vision-related quality of life (Mangione et al. 1998). Growing evidence suggests that reading difficulties, such as slow reading or reading fatigue, are common among individuals with bilateral glaucoma (Ramulu et al. 2013), even in moderate stages of the disease (Nelson et al. 1999, 2003). These challenges are particularly notable when reading small print, low-contrast text, lengthy passages, or in dimly lit environments (Altangerel et al. 2006, Kulkarni et al. 2012, Ramulu et al. 2013).

Reading difficulties have been identified as a primary complaint among individuals with glaucoma (Aspinall et al. 2008, Fujita et al. 2006, McKean-Cowdin et al. 2008, Stamper 1984, Viswanathan et al. 1999). For instance, Duke-Elder (1969) cited a survey conducted by the Glaucoma Clinic at the University of London showing that 66% of patients reported experiencing blurred vision or challenges in reading. Moreover, patients consistently mentioned difficulties with reading, following lines of text (Viswanathan et al. 1999), and completing other near-vision tasks (Freeman et al. 2008, Nelson et al. 1999) in interview and questionnaire studies. In addition, Nguyen et al. (2014) investigated the impact of glaucoma-related vision loss on reading abilities across a broad range of reading tasks by evaluating reading engagement through weekly reading activity assessments. They observed significant reductions in reading abilities among glaucoma patients compared to healthy controls (*p* < 0.05). Specifically, for each 5-dB decrement in the better eye’s MD, there was an associated 18% reduction in days spent on book reading and a 10% decrease in days spent on reading newspapers. These declines were linked to more severe visual field loss and decreased contrast sensitivity within the glaucoma patient group.

Consistent with patients’ self-reported difficulties in reading, objective assessments of both oral and silent reading further confirm slower reading speeds in individuals with bilateral glaucoma compared to age-matched healthy controls (Crabb et al. 2013; Mathews et al. 2015; Ramulu et al. 2009, 2013; Smith et al. 2014). As slow and effortful reading in impaired vision often reflects a bottom-up, visual sensory limitation on reading, reading speed has been a functionally significant measure (Legge 2006). In a study by Ikeda et al. (2021), the MNREAD test was conducted on 35 glaucoma patients (MD: −6.29 dB and −11.08 dB for the better and worse eyes, respectively) and 32 similarly aged controls with visual acuity better than 0.4 logMAR in both eyes. Glaucoma patients exhibited significantly slower reading speeds, with an average of 83 compared to an average of 102 words per minute in healthy controls (*p* < 0.01). Similarly, Kwon et al. (2017) found that glaucoma patients (MD: −6.23 dB and −12.09 dB for the better and worse eyes, respectively) with normal binocular visual acuity (20/20 Snellen equivalent) exhibit significantly slower oral reading speed (20% slower, *p* < 0.05) compared to healthy controls, even after controlling for age. These findings collectively suggest that, despite having relatively normal visual acuity, individuals with moderate glaucoma exhibit poor reading performance compared to age-similar healthy controls.

Importantly, reading difficulties are further exacerbated when dealing with small print (Altangerel et al. 2006), low-contrast text (Burton et al. 2012), prolonged reading periods (Ramulu et al. 2013), or low-light conditions (Goddin et al. 2023). For example, Burton et al. (2012) showed that glaucoma patients exhibited a significant reduction in reading speed, particularly as text contrast levels decreased from 100% to 20%, compared to age-matched normal cohorts. The significant association between contrast sensitivity and reading speed was also reported in a study by Ramulu et al. (2013). In addition, Altangerel et al. (2006) identified the reading of small print as one of the most visually demanding tasks for glaucoma patients and noted a moderate correlation between reading speed and the extent of binocular visual field loss. Furthermore, anomalies in the pattern of eye movements during reading have been observed in glaucoma patients; these anomalies may contribute to their reading difficulties. For example, Smith et al. (2014) examined the pattern of eye movements during reading by individuals with glaucoma by comparing reading done by the better and the worse eye using an eye tracker. Their findings showed significant differences in reading speed and saccade rates between the two eyes. Specifically, slower reading with the worse eye was associated with a higher occurrence of regressive saccades. It is also important to note that specific features, such as the number of letters, word frequency, and the location of a word at the end of a line of text, seem to pose additional challenges for individuals with glaucoma when reading (Mathews et al. 2015). On the other hand, Goddin et al. (2023) investigated the impact of low luminance on reading abilities in individuals with early and moderate stages of glaucoma. Reading vision was assessed using the MNREAD test under both mesopic (2 cd/m^2^) and photopic (220 cd/m^2^) conditions. They found that, compared to healthy controls, glaucoma patients exhibited worse reading vision irrespective of the viewing condition, as indicated by slower reading speed, reduced reading accessibility, and a need for larger print sizes. Moreover, the difference between the two groups became significantly greater under low-luminance conditions (*p* < 0.05), even after controlling for age and visual acuity. The findings suggest that reading in dim light poses greater challenges for individuals with glaucoma despite their normal photopic visual acuity. This further supports the notion that visual acuity alone may not be the best predictor for functional reading vision. Given the importance of reading in everyday life, reading tests could offer a more comprehensive evaluation of a patient’s functional vision. They could also serve as potentially useful tools for assessing the efficacy of interventions and monitoring the progression of the disease.

It is apparent that various visual and perceptual factors collectively contribute to reading difficulties in glaucoma. Extensive evidence has shown that a decrease in the visual span, largely limited by crowding, coupled with deficiencies in letter recognition, such as reduced acuity or contrast sensitivity, significantly impede reading speed (Legge et al. 1985). Therefore, it is plausible to infer that the combination of reduced contrast sensitivity and increased crowding in the central visual field of glaucomatous vision might jointly contribute to the shrinkage of the visual span and functional field of view. This, in turn, may lead to reading difficulty in glaucoma, as illustrated in Figure 3. Potentially similar mechanisms could underlie central vision deficits in face or object recognition in individuals with glaucoma. While progress has been made in understanding how glaucoma affects tasks relying on central vision, there is still a need for further research to unravel the perceptual and cortical mechanisms responsible for central vision deficits in glaucoma.

## 5. CONCLUDING REMARKS

Converging evidence indicates that even early glaucomatous damage involves the macula more commonly than has been previously thought. Loss or dysfunction of RGCs in the macular region is closely related to central vision deficits in individuals with glaucoma, thereby affecting the quality of life. It is thus important to gain a better understanding of how glaucomatous damage affects perceptual processes related to central vision tasks like reading and object or face recognition, typically not considered to be affected until the late stages of the disease. In this review, I discuss how RGC damage or dysfunction may impact central pattern vision by reviewing the fundamental role of RGCs in contrast coding and spatial summation of visual input. It is evident that glaucomatous damage in both the central and peripheral visual fields contributes to difficulties in performing daily visual activities in individuals with glaucoma. Thus, future research is called for to unravel the perceptual and cortical mechanisms that underlie deficits in both central and peripheral vision across the different stages of glaucoma.

## SUMMARY POINTS

1. Among their various functions, retinal ganglion cells (RGCs) perform key computations necessary for contrast coding and spatial summation, which are fundamental for human pattern vision.
2. Ricco’s law and Piper’s law encapsulate the inverse relationship between threshold contrast and target size (or summation area), which is often overlooked in routine visual sensitivity assessments where one aspect (size or contrast) is fixed while the other is measured.
3. Variation in human visual sensitivity across the visual field, including contrast sensitivity and the critical area of spatial summation (e.g., Ricco’s area), is closely linked to the topographic inhomogeneity of RGC density across the retina.
4. A constant number of RGCs have been shown to subserve the critical area of spatial summation across the visual field. Loss of RGCs is linked to a reduction in contrast sensitivity and an enlargement in the critical area of spatial summation.
5. Advancements in retinal imaging techniques, such as optical coherence tomography, have revealed notable macular damage in early stages of glaucoma. Consistent with structural damage, both subjective reports and objective functional assessments have indicated central visual dysfunction in glaucoma.
6. Glaucomatous vision shows reduced contrast sensitivity and increased crowding in the central visual field, which appears to collectively contribute to the reduction of the visual span and the functional field of view in glaucoma.
7. The decrease in both the visual span and the functional field of view significantly impacts central vision tasks, such as reading and object or face recognition, especially under higher sensory or cognitive demands.
8. Conventional assessments, such as high-contrast single-letter recognition for visual acuity and standard perimetry reliant on spot-light detection against a uniform background, might not fully represent a patient’s functional vision necessary for everyday tasks.

## FUTURE ISSUES

1. Glaucoma patients are more susceptible to tasks involving higher cognitive demands. Therefore, it is important to characterize the impact of glaucomatous damage on higher-level cognitive processes, such as attention or working memory.
2. Glaucomatous damage is often binocularly asymmetric, as well as monocularly inhomogeneous, across the visual field. How does the visual system cope with such asymmetric and inhomogeneous visual field defects? How do such visual field defects affect oculomotor behavior, visuomotor coordination, or neural processing?
3. How does glaucomatous damage interact with low-luminance or mesopic light conditions? What are the mechanisms underlying the additional deficits caused by mesopic conditions in glaucoma?
4. Many glaucoma patients are often unaware of their visual field loss (Crabb et al. 2013), which has been attributed to a perceptual phenomenon known as filling-in. Through this process, the visual system fills in the missing information with surrounding visual attributes. The lack of scotoma awareness presents a challenge for early detection and intervention. Are there effective methods to enhance scotoma awareness in glaucoma patients?
5. Although ongoing treatment may slow down glaucoma progression, individuals with existing visual field loss must cope with compromised vision in daily activities. What effective rehabilitative strategies would optimize the remaining vision to improve everyday visual performance?

## References

[R1] Altangerel U, Spaeth GL, Steinmann WC. 2006. Assessment of Function Related to Vision (AFREV). Ophthalmic Epidemiol. 13:67–80 16510349 10.1080/09286580500428500

[R2] Aspinall PA, Johnson ZK, Azuara-Blanco A, Montarzino A, Brice R, Vickers A. 2008. Evaluation of quality of life and priorities of patients with glaucoma. Investig. Ophthalmol. Vis. Sci. 49:1907–15 18436824 10.1167/iovs.07-0559

[R3] Avidan G, Harel M, Hendler T, Ben-Bashat D, Zohary E, Malach R. 2002. Contrast sensitivity in human visual areas and its relationship to object recognition. J. Neurophysiol. 87:3102–16 12037211 10.1152/jn.2002.87.6.3102

[R4] Ball K, Owsley C. 1993. The useful field of view test: a new technique for evaluating age-related declines in visual function. J. Am. Optom. Assoc. 64:71–99 8454831

[R5] Ball K, Owsley C, Sloane ME, Roenker DL, Bruni JR.1993.Visual attention problems as a predictor of vehicle crashes in older drivers. Investig. Ophthalmol. Vis. Sci. 34:3110–23 8407219

[R6] Ball KK, Beard BL, Roenker DL, Miller RL, Griggs DS. 1988. Age and visual search: expanding the useful field of view. J. Opt. Soc. Am. A 5:2210–19 3230491 10.1364/josaa.5.002210

[R7] Bambo MP, Ferrandez B, Güerri N, Fuertes I, Cameo B, 2016. Evaluation of contrast sensitivity, chromatic vision, and reading ability in patients with primary open angle glaucoma. J. Ophthalmol. 2016:7074016 27872754 10.1155/2016/7074016 PMC5107829

[R8] Barlow HB. 1953. Summation and inhibition in the frog’s retina. J. Physiol. 119:69–88 13035718 10.1113/jphysiol.1953.sp004829 PMC1393035

[R9] Barlow HB. 1958. Temporal and spatial summation in human vision at different background intensities. J. Physiol. 141:337–50 13539843 10.1113/jphysiol.1958.sp005978 PMC1358805

[R10] Barlow HB, Fitzhugh R, Kuffler SW. 1957. Change of organization in the receptive fields of the cat’s retina during dark adaptation. J. Physiol. 137:338–54 13463771 10.1113/jphysiol.1957.sp005817 PMC1363009

[R11] Bhorade AM, Perlmutter MS, Wilson B, Kambarian J, Chang S, 2013. Differences in vision between clinic and home and the effect of lighting in older adults with and without glaucoma. JAMA Ophthalmol. 131:1554–62 24263699 10.1001/jamaophthalmol.2013.4995 PMC4377300

[R12] Bicket AK, Mihailovic A, E J-Y, Nguyen A, Mukherjee MR, 2020. Gait in elderly glaucoma: impact of lighting conditions, changes in lighting, and fear of falling. Transl. Vis. Sci. Technol. 9:23 10.1167/tvst.9.13.23 PMC7745602 33364078

[R13] Bierings RAJM, Overkempe T, van Berkel CM, Kuiper M, Jansonius NM. 2019. Spatial contrast sensitivity from star- to sunlight in healthy subjects and patients with glaucoma. Vis. Res. 158:31–39 30721742 10.1016/j.visres.2019.01.011

[R14] Blumberg DM, Liebmann JM, Hirji SH, Hood DC. 2019. Diffuse macular damage in mild to moderate glaucoma is associated with decreased visual function scores under low luminance conditions. Am. J. Ophthalmol. 208:415–20 31493403 10.1016/j.ajo.2019.08.024

[R15] Bonneh YS, Sagi D, Polat U. 2007. Spatial and temporal crowding in amblyopia. Vis. Res. 47:1950–62 17502115 10.1016/j.visres.2007.02.015

[R16] Bosworth CF, Sample PA, Weinreb RN. 1997. Motion perception thresholds in areas of glaucomatous visual field loss. Vis. Res. 37:355–64 9135868 10.1016/s0042-6989(96)00136-8

[R17] Bouma H 1970. Interaction effects in parafoveal letter recognition. Nature 226:177–78 5437004 10.1038/226177a0

[R18] Bullimore MA, Wood JM, Swenson K. 1993. Motion perception in glaucoma. Investig. Ophthalmol. Vis. Sci. 34:3526–33 8258510

[R19] Burton R, Crabb DP, Smith ND, Glen FC, Garway-Heath DF. 2012. Glaucoma and reading: exploring the effects of contrast lowering of text. Optom. Vis. Sci. 89:1282–87 22885786 10.1097/OPX.0b013e3182686165

[R20] Byrne JH. 1997. Neuroscience Online: An Electronic Textbook for the Neurosciences. Houston, TX: McGovern Med. School UTHealth

[R21] Carandini M, Heeger DJ. 2011. Normalization as a canonical neural computation. Nat. Rev. Neurosci. 13:51–62 22108672 10.1038/nrn3136 PMC3273486

[R22] Cavanaugh JR, Bair W, Movshon JA. 2002. Nature and interaction of signals from the receptive field center and surround in macaque V1 neurons. J. Neurophysiol. 88:2530–46 12424292 10.1152/jn.00692.2001

[R23] Chauhan BC, House PH, McCormick TA, Leblanc RP. 1999. Comparison of conventional and high-pass resolution perimetry in a prospective study of patients with glaucoma and healthy controls. Arch. Ophthalmol. 117:24–33 9930157 10.1001/archopht.117.1.24

[R24] Chauhan BC, Pan J, Archibald ML, Levatte TL, Kelly MEM, Tremblay FO. 2002. Effect of intraocular pressure on optic disc topography, electroretinography, and axonal loss in a chronic pressure-induced rat model of optic nerve damage. Investig. Ophthalmol. Vis. Sci. 43:2969–76 12202517

[R25] Cheong AM, Legge GE, Lawrence MG, Cheung SH, Ruff MA. 2008. Relationship between visual span and reading performance in age-related macular degeneration. Vis. Res. 48:577–88 18191983 10.1016/j.visres.2007.11.022 PMC2323914

[R26] Chien L, Liu R, Girkin C, Kwon M. 2017. Higher contrast requirement for letter recognition and macular RGC+ layer thinning in glaucoma patients and older adults. Investig. Ophthalmol. Vis. Sci. 58:6221–31 29228250 10.1167/iovs.17-22621 PMC5724554

[R27] Cowan CS, Sabharwal J, Seilheimer RL, Wu SM. 2017. Distinct subcomponents of mouse retinal ganglion cell receptive fields are differentially altered by light adaptation. Vis. Res. 131:96–105 28087445 10.1016/j.visres.2016.12.015

[R28] Cowey A, Rolls ET. 1974. Human cortical magnification factor and its relation to visual acuity. Exp. Brain Res. 21:447–54 4442497 10.1007/BF00237163

[R29] Crabb DP. 2016. A view on glaucoma—are we seeing it clearly? Eye 30:304–13 26611846 10.1038/eye.2015.244 PMC4763130

[R30] Crabb DP, Smith ND, Glen FC, Burton R, Garway-Heath DF. 2013. How does glaucoma look? Patient perception of visual field loss. Ophthalmology 120:1120–26 23415421 10.1016/j.ophtha.2012.11.043

[R31] Curcio CA, Allen KA. 1990. Topography of ganglion cells in human retina. J. Comp. Neurol. 300:5–25 2229487 10.1002/cne.903000103

[R32] de A Moura AL, Raza AS, Lazow MA, de Moraes CG, Hood DC. 2012. Retinal ganglion cell and inner plexiform layer thickness measurements in regions of severe visual field sensitivity loss in patients with glaucoma. Eye 26:1188–93 22699978 10.1038/eye.2012.110 PMC3443822

[R33] de Valois RL, Morgan H, Snodderly DM. 1974. Psychophysical studies of monkey vision. 3. Spatial luminance contrast sensitivity tests of macaque and human observers. Vis. Res. 14:75–81 4204839 10.1016/0042-6989(74)90118-7

[R34] Dougherty RF, Koch VM, Brewer AA, Fischer B, Modersitzki J, Wandell BA. 2003. Visual field representations and locations of visual areas V1/2/3 in human visual cortex. J. Vis. 3:586–98 14640882 10.1167/3.10.1

[R35] Drasdo N, Millican CL, Katholi CR, Curcio CA. 2007. The length of Henle fibers in the human retina and a model of ganglion receptive field density in the visual field. Vis. Res. 47:2901–11 17320143 10.1016/j.visres.2007.01.007 PMC2077907

[R36] Duke-Elder S 1969. Diseases of the lens and vitreous: glaucoma and hypotony. In System of Ophthalmology, Vol. XI, p. 443. London: Henry Kimpton

[R37] Dumoulin SO, Wandell BA. 2008. Population receptive field estimates in human visual cortex. NeuroImage 39:647–60 17977024 10.1016/j.neuroimage.2007.09.034 PMC3073038

[R38] Duncan RO, Boynton GM. 2003. Cortical magnification within human primary visual cortex correlates with acuity thresholds. Neuron 38:659–71 12765616 10.1016/s0896-6273(03)00265-4

[R39] E J-Y, Mihailovic A, Garzon C, Schrack JA, Li T, 2021. Association between visual field damage and gait dysfunction in patients with glaucoma. JAMA Ophthalmol. 139:1053–60 34292297 10.1001/jamaophthalmol.2021.2617 PMC8299359

[R40] el-Gohary A, Siam G. 2009. Stereopsis and contrast sensitivity binocular summation in early glaucoma. Res. J. Med. Med. Sci. 4:85–88

[R41] Engel SA, Glover GH, Wandell BA. 1997. Retinotopic organization in human visual cortex and the spatial precision of functional MRI. Cereb. Cortex 7:181–92 9087826 10.1093/cercor/7.2.181

[R42] Enroth-Cugell C, Robson JG. 1966. The contrast sensitivity of retinal ganglion cells of the cat. J. Physiol. 187:517–52 16783910 10.1113/jphysiol.1966.sp008107 PMC1395960

[R43] Fellman RL, Lynn JR, Starita RJ, Swanson WH. 1989. Clinical Importance of Spatial Summation in Glaucoma. Amsterdam: Kugler Gedini

[R44] Fileta JB, Huang W, Kwon GP, Filippopoulos T, Ben Y, 2008. Efficient estimation of retinal ganglion cell number: a stereological approach. J. Neurosci. Methods 170:1–8 18241929 10.1016/j.jneumeth.2007.12.008 PMC2875943

[R45] Fischer B 1973. Overlap of receptive field centers and representation of the visual field in the cat’s optic tract. Vis. Res. 13:2113–20 4763524 10.1016/0042-6989(73)90188-0

[R46] Freed M, Sterling P. 1988. The ON-alpha ganglion cell of the cat retina and its presynaptic cell types. J. Neurosci. 8:2303–20 3249227 10.1523/JNEUROSCI.08-07-02303.1988 PMC6569538

[R47] Freeman EE, Munoz B, West SK, Jampel HD, Friedman DS. 2008. Glaucoma and quality of life: the Salisbury Eye Evaluation. Ophthalmology 115:233–38 17655930 10.1016/j.ophtha.2007.04.050

[R48] Friedman DS, Freeman E, Munoz B, Jampel HD, West SK. 2007. Glaucoma and mobility performance: the Salisbury Eye Evaluation Project. Ophthalmology 114:2232–37 17980433 10.1016/j.ophtha.2007.02.001

[R49] Fujita K, Yasuda N, Oda K, Yuzawa M. 2006. Reading performance in patients with central visual field disturbance due to glaucoma. Nippon Ganka Gakkai Zasshi 110:914–18 17134038

[R50] Garway-Heath DF, Caprioli J, Fitzke FW, Hitchings RA. 2000. Scaling the hill of vision: the physiological relationship between light sensitivity and ganglion cell numbers. Investig. Ophthalmol. Vis. Sci 41:1774–82 10845598

[R198] Gattass R, Gross CG, Sandell JH. 1981. Visual topography of V2 in the macaque. J. Comp. Neurol. 201:519–39 7287933 10.1002/cne.902010405

[R51] Gilbert CD, Hirsch JA, Wiesel TN. 1990. Lateral interactions in visual cortex. Cold Spring Harb. Symp. Quant. Biol. 55:663–77 2132846 10.1101/sqb.1990.055.01.063

[R52] Glen FC, Crabb DP, Smith ND, Burton R, Garway-Heath DF. 2012. Do patients with glaucoma have difficulty recognizing faces? Investig. Ophthalmol. Vis. Sci. 53:3629–37 22511628 10.1167/iovs.11-8538

[R53] Glen FC, Smith ND, Crabb DP. 2013. Saccadic eye movements and face recognition performance in patients with central glaucomatous visual field defects. Vis. Res. 82:42–51 23485426 10.1016/j.visres.2013.02.010

[R54] Glovinsky Y, Quigley HA, Pease ME. 1993. Foveal ganglion cell loss is size dependent in experimental glaucoma. Investig. Ophthalmol. Vis. Sci. 34:395–400 8440594

[R55] Goddin T-L, Yu H, Friedman DS, Owsley C, Kwon M. 2023. MNREAD reading vision in adults with glaucoma under mesopic and photopic conditions. Investig. Ophthalmol. Vis. Sci. 64:43 10.1167/iovs.64.15.43 PMC10756241 38153749

[R56] Greenfield DS, Bagga H, Knighton RW. 2003. Macular thickness changes in glaucomatous optic neuropathy detected using optical coherence tomography. Arch. Ophthalmol. 121:41–46 12523883 10.1001/archopht.121.1.41

[R57] Grüsser OJ. 1995. Migraine phosphenes and the retino-cortical magnification factor. Vis. Res. 35:1125–34 7762167 10.1016/0042-6989(94)00187-q

[R58] Hariharan S, Levi DM, Klein SA. 2005. “Crowding” in normal and amblyopic vision assessed with Gaussian and Gabor C’s. Vis. Res. 45:617–33 15621179 10.1016/j.visres.2004.09.035

[R59] Harwerth RS, Carter-Dawson L, Shen F, Smith EL 3rd, Crawford ML. 1999. Ganglion cell losses underlying visual field defects from experimental glaucoma. Investig. Ophthalmol. Vis. Sci. 40:2242–50 10476789

[R60] Harwerth RS, Carter-Dawson L, Smith EL 3rd, Barnes G, Holt WF, Crawford ML. 2004. Neural losses correlated with visual losses in clinical perimetry. Investig. Ophthalmol. Vis. Sci. 45:3152–60 15326134 10.1167/iovs.04-0227

[R61] Harwerth RS, Wheat JL, Fredette MJ, Anderson DR. 2010. Linking structure and function in glaucoma. Prog. Retin. Eye Res. 29:249–71 20226873 10.1016/j.preteyeres.2010.02.001 PMC2878911

[R62] Hawkins AS, Szlyk JP, Ardickas Z, Alexander KR, Wilensky JT.2003.Comparison of contrast sensitivity, visual acuity, and Humphrey visual field testing in patients with glaucoma. J. Glaucoma 12:134–38 12671468

[R63] Haymes SA, Leblanc RP, Nicolela MT, Chiasson LA, Chauhan BC. 2007. Risk of falls and motor vehicle collisions in glaucoma. Investig. Ophthalmol. Vis. Sci. 48:1149–55 17325158 10.1167/iovs.06-0886

[R64] Haymes SA, Leblanc RP, Nicolela MT, Chiasson LA, Chauhan BC. 2008. Glaucoma and on-road driving performance. Investig. Ophthalmol. Vis. Sci. 49:3035–41 18326696 10.1167/iovs.07-1609

[R65] He S, Cavanagh P, Intriligator J. 1996. Attentional resolution and the locus of visual awareness. Nature 383:334–37 8848045 10.1038/383334a0

[R66] Hertenstein H, Bach M, Gross NJ, Beisse F. 2016. Marked dissociation of photopic and mesopic contrast sensitivity even in normal observers. Graefes Arch. Clin. Exp. Ophthalmol. 254:373–84 25921390 10.1007/s00417-015-3020-4

[R67] Hirji SH, Hood DC, Liebmann JM, Blumberg DM. 2021. Association of patterns of glaucomatous macular damage with contrast sensitivity and facial recognition in patients with glaucoma. JAMA Ophthalmol. 139:27–32 33151275 10.1001/jamaophthalmol.2020.4749 PMC7645734

[R68] Hood DC, Raza AS, de Moraes CG, Johnson CA, Liebmann JM, Ritch R. 2012. The nature of macular damage in glaucoma as revealed by averaging optical coherence tomography data. Transl. Vis. Sci. Technol. 1:3 10.1167/tvst.1.1.3 PMC3634586 23626924

[R69] Hood DC, Raza AS, de Moraes CG, Liebmann JM, Ritch R. 2013. Glaucomatous damage of the macula. Prog. Retin Eye Res. 32:1–21 22995953 10.1016/j.preteyeres.2012.08.003 PMC3529818

[R70] Hood DC, Raza AS, de Moraes CG, Odel JG, Greenstein VC, 2011. Initial arcuate defects within the central 10 degrees in glaucoma. Investig. Ophthalmol. Vis. Sci. 52:940–46 20881293 10.1167/iovs.10-5803 PMC3053114

[R71] Hood DC, Slobodnick A, Raza AS, de Moraes CG, Teng CC, Ritch R. 2014. Early glaucoma involves both deep local, and shallow widespread, retinal nerve fiber damage of the macular region. Investig. Ophthalmol. Vis. Sci. 55:632–49 24370831 10.1167/iovs.13-13130 PMC3912939

[R72] Horn F, Martus P, Korth M. 1995. Comparison of temporal and spatiotemporal contrast-sensitivity tests in normal subjects and glaucoma patients. German J. Ophthalmol. 4:97–102 7795517

[R73] Horton JC, Hoyt WF. 1991. The representation of the visual field in human striate cortex. A revision of the classic Holmes map. Arch. Ophthalmol. 109:816–24 2043069 10.1001/archopht.1991.01080060080030

[R74] Howarth CI, Lowe G. 1966. Statistical detection theory of Piper’s law. Nature 212:324–26 5970140 10.1038/212324a0

[R75] Huang D, Swanson EA, Lin CP, Schuman JS, Stinson WG, 1991.Optical coherence tomography. Science 254:1178–81 1957169 10.1126/science.1957169 PMC4638169

[R76] Huisingh C, McGwin G, Wood J, Owsley C. 2015. The driving visual field and a history of motor vehicle collision involvement in older drivers: a population-based examination. Investig. Ophthalmol. Vis. Sci. 56:132–38 10.1167/iovs.14-15194 PMC4288142 25395488

[R77] Ichhpujani P, Thakur S, Spaeth GL. 2020. Contrast sensitivity and glaucoma. J. Glaucoma 29:71–75 31567752 10.1097/IJG.0000000000001379

[R78] Ikeda MC, Bando AH, Hamada KU, Nakamura VPL, Prata TS, 2021. Is reading performance impaired in glaucoma patients with preserved central vision? J. Glaucoma 30:e153–58 33534509 10.1097/IJG.0000000000001806

[R79] Inui T, Mimura O, Kani K. 1981. Retinal sensitivity and spatial summation in the foveal and parafoveal regions. J. Opt. Soc. Am. 71:151–63 7277059 10.1364/josa.71.000151

[R80] Issashar Leibovitzh G, Trope GE, Kherani IN, Buys YM, Tarita-Nistor L. 2023. Atypical responses to faces during binocular rivalry in early glaucoma. Front. Neurosci. 17:1151278 37304026 10.3389/fnins.2023.1151278 PMC10248174

[R81] Je S, Ennis FA, Woodhouse JM, Sengpiel F, Redmond T. 2018. Spatial summation across the visual field in strabismic and anisometropic amblyopia. Sci. Rep. 8:3858 29497120 10.1038/s41598-018-21620-6 PMC5832776

[R82] Joffe KM, Raymond JE, Chrichton A. 1997. Motion coherence perimetry in glaucoma and suspected glaucoma. Vis. Res. 37:955–64 9156192 10.1016/s0042-6989(96)00221-0

[R83] Kay KN, Winawer J, Mezer A, Wandell BA. 2013. Compressive spatial summation in human visual cortex. J. Neurophysiol. 110:481–94 23615546 10.1152/jn.00105.2013 PMC3727075

[R84] Kelly DH. 1975. Spatial frequency selectivity in the retina. Vis. Res. 15:665–72 1138482 10.1016/0042-6989(75)90282-5

[R85] Kerrigan-Baumrind LA, Quigley HA, Pease ME, Kerrigan DF, Mitchell RS. 2000. Number of ganglion cells in glaucoma eyes compared with threshold visual field tests in the same persons. Investig. Ophthalmol. Vis. Sci. 41:741–48 10711689

[R86] King WM, Sarup V, Sauve Y, Moreland CM, Carpenter DO, Sharma SC. 2006. Expansion of visual receptive fields in experimental glaucoma. Vis. Neurosci. 23:137–42 16597357 10.1017/S0952523806231122

[R87] King-Smith PE, Carden D. 1976. Luminance and opponent-color contributions to visual detection and adaptation and to temporal and spatial integration. J. Opt. Soc. Am. 66:709–17 978286 10.1364/josa.66.000709

[R88] Klein J, Pierscionek BK, Lauritzen J, Derntl K, Grzybowski A, Zlatkova MB. 2015. The effect of cataract on early stage glaucoma detection using spatial and temporal contrast sensitivity tests. PLOS ONE 10:e0128681 26053793 10.1371/journal.pone.0128681 PMC4460016

[R89] Kotecha A, O’Leary N, Melmoth D, Grant S, Crabb DP. 2009. The functional consequences of glaucoma for eye-hand coordination. Investig. Ophthalmol. Vis. Sci. 50:203–13 18806294 10.1167/iovs.08-2496

[R90] Kulkarni KM, Mayer JR, Lorenzana LL, Myers JS, Spaeth GL. 2012. Visual field staging systems in glaucoma and the activities of daily living. Am. J. Ophthalmol. 154:445–51.e3 22633358 10.1016/j.ajo.2012.03.030

[R91] Kwon M, Huisingh C, Rhodes LA, McGwin G Jr., Wood JM, Owsley C. 2016. Association between glaucoma and at-fault motor vehicle collision involvement among older drivers: a population-based study. Ophthalmology 123:109–16 26459997 10.1016/j.ophtha.2015.08.043 PMC4695303

[R92] Kwon M, Legge GE, Dubbels BR. 2007. Developmental changes in the visual span for reading. Vis. Res. 47:2889–900 17845810 10.1016/j.visres.2007.08.002 PMC2052928

[R93] Kwon M, Liu R. 2019. Linkage between retinal ganglion cell density and the nonuniform spatial integration across the visual field. PNAS 116:3827–36 30737290 10.1073/pnas.1817076116 PMC6397585

[R94] Kwon M, Liu R. 2021. Identifying and localizing retinal features that predict human contrast sensitivity via deep learning. J. Vis. 21:2615 10.1167/iovs.63.2.27 PMC8859491 35179554

[R95] Kwon M, Liu R, Patel BN, Girkin C. 2017. Slow reading in glaucoma: Is it due to the shrinking visual span in central vision? Investig. Ophthalmol. Vis. Sci. 58:5810–18 29131903 10.1167/iovs.17-22560 PMC5808572

[R96] Lahav K, Levkovitch-Verbin H, Belkin M, Glovinsky Y, Polat U. 2011. Reduced mesopic and photopic foveal contrast sensitivity in glaucoma. Arch. Ophthalmol. 129:16–22 21220624 10.1001/archophthalmol.2010.332

[R97] Larson AM, Loschky LC. 2009. The contributions of central versus peripheral vision to scene gist recognition. J. Vis. 9:6 19810787 10.1167/9.10.6

[R98] Leat SJ, Woodhouse JM. 1993. Reading performance with low vision aids: relationship with contrast sensitivity. Ophthalmic Physiol. Opt. 13:9–16 8510953 10.1111/j.1475-1313.1993.tb00420.x

[R99] Lee SS, Black AA, Wood JM. 2018. Scanning behavior and daytime driving performance of older adults with glaucoma. J. Glaucoma 27:558–65 29613977 10.1097/IJG.0000000000000962

[R100] Lee SSY, Wood JM, Black AA. 2020. Impact of glaucoma on executive function and visual search. Ophthalmic Physiol. Opt. 40:333–42 32189400 10.1111/opo.12679

[R101] Legge GE. 2006. Psychophysics of Reading in Normal and Low Vision. Abingdon: Taylor & Francis

[R102] Legge GE, Ahn SJ, Klitz TS, Luebker A. 1997. Psychophysics of reading. XVI. The visual span in normal and low vision. Vis. Res. 37:1999–2010 9274784 10.1016/s0042-6989(97)00017-5

[R103] Legge GE, Mansfield JS, Chung ST.2001.Psychophysics of reading. XX. Linking letter recognition to reading speed in central and peripheral vision. Vis. Res. 41:725–43 11248262 10.1016/s0042-6989(00)00295-9

[R104] Legge GE, Rubin GS, Pelli DG, Schleske MM. 1985. Psychophysics of reading. II. Low vision. Vis.Res.25:253–65 4013092 10.1016/0042-6989(85)90118-x

[R105] Lenoble Q, Lek JJ, McKendrick AM. 2016. Visual object categorisation in people with glaucoma. Br. J. Ophthalmol. 100:1585–90 27503395 10.1136/bjophthalmol-2015-308289

[R106] Lin S, Mihailovic A, West SK, Johnson CA, Friedman DS, 2018. Predicting visual disability in glaucoma with combinations of vision measures. Transl. Vis. Sci. Technol. 7:22 10.1167/tvst.7.2.22 PMC5901371 29670831

[R107] Liu R, Kwon M. 2020. Increased equivalent input noise in glaucomatous central vision: Is it due to undersampling of retinal ganglion cells? Investig. Ophthalmol. Vis. Sci. 61:10 10.1167/iovs.61.8.10 PMC7425734 32645132

[R108] Liu R, Patel BN, Kwon M. 2017a. Age-related changes in crowding and reading speed. Sci. Rep. 7:8271 28811585 10.1038/s41598-017-08652-0 PMC5557829

[R109] Liu Z, Kurokawa K, Zhang F, Lee JJ, Miller DT. 2017b. Imaging and quantifying ganglion cells and other transparent neurons in the living human retina. PNAS 114:12803–8 29138314 10.1073/pnas.1711734114 PMC5715765

[R110] Mangione CM, Berry S, Spritzer K, Janz NK, Klein R, 1998. Identifying the content area for the 51-item National Eye Institute Visual Function Questionnaire: results from focus groups with visually impaired persons. Arch. Ophthalmol. 116:227–33 9488276 10.1001/archopht.116.2.227

[R111] Marr D, Hildreth E. 1980. Theory of edge detection. Proc. R. Soc. B 207:187–217 6102765 10.1098/rspb.1980.0020

[R112] Mathews PM, Rubin GS, McCloskey M, Salek S, Ramulu PY. 2015. Severity of vision loss interacts with word-specific features to impact out-loud reading in glaucoma. Investig. Ophthalmol. Vis. Sci. 56:1537–45 25737150 10.1167/iovs.15-15462 PMC4349108

[R113] McConkie GW, Rayner K. 1975. The span of the effective stimulus during a fixation in reading. Percept. Psychophys. 17:578–86

[R114] McKean-Cowdin R, Wang Y, Wu J, Azen SP, Varma R, 2008. Impact of visual field loss on health-related quality of life in glaucoma: the Los Angeles Latino Eye Study. Ophthalmology 115:941–48.e1 17997485 10.1016/j.ophtha.2007.08.037 PMC4864605

[R115] McKendrick AM, Sampson GP, Walland MJ, Badcock DR. 2007. Contrast sensitivity changes due to glaucoma and normal aging: low-spatial-frequency losses in both magnocellular and parvocellular pathways. Investig. Ophthalmol. Vis. Sci. 48:2115–22 17460269 10.1167/iovs.06-1208

[R116] Mihailovic A, Swenor BK, Friedman DS, West SK, Gitlin LN, Ramulu PY. 2017. Gait implications of visual field damage from glaucoma. Transl. Vis. Sci. Technol. 6:23 28660098 10.1167/tvst.6.3.23 PMC5484170

[R117] Moes E, Lombardi KM. 2009. The relationship between contrast sensitivity, gait, and reading speed in Parkinson’s disease. Neuropsychol. Dev. Cogn. B 16:121–32 10.1080/13825580802233418 18688759

[R118] Mulholland PJ, Redmond T, Garway-Heath DF, Zlatkova MB, Anderson RS. 2015. Spatiotemporal summation of perimetric stimuli in early glaucoma. Investig. Ophthalmol. Vis. Sci. 56:6473–82 26447981 10.1167/iovs.15-16921

[R119] Nelson P, Aspinall P, O’Brien C. 1999. Patients’ perception of visual impairment in glaucoma: a pilot study. Br. J. Ophthalmol. 83:546–52 10216052 10.1136/bjo.83.5.546 PMC1723044

[R120] Nelson P, Aspinall P, Papasouliotis O, Worton B, O’Brien C. 2003. Quality of life in glaucoma and its relationship with visual function. J. Glaucoma 12:139–50 12671469 10.1097/00061198-200304000-00009

[R121] Nguyen AM, van Landingham SW, Massof RW, Rubin GS, Ramulu PY. 2014. Reading ability and reading engagement in older adults with glaucoma. Investig. Ophthalmol. Vis. Sci. 55:5284–90 25052992 10.1167/iovs.14-14138 PMC4142770

[R122] Ogata NG, Boer ER, Daga FB, Jammal AA, Stringham JM, Medeiros FA. 2019. Visual crowding in glaucoma. Investig. Ophthalmol. Vis. Sci. 60:538–43 30716149 10.1167/iovs.18-25150 PMC6361551

[R123] Owsley C 2003. Contrast sensitivity. Ophthalmol. Clin. North Am. 16:171–77 12809156 10.1016/s0896-1549(03)00003-8

[R124] Owsley C 2013. Visual processing speed. Vis. Res. 90:52–56 23231958 10.1016/j.visres.2012.11.014 PMC3615057

[R125] Owsley C, McGwin G Jr. 1999. Vision impairment and driving. Surv. Ophthalmol. 43:535–50 10416796 10.1016/s0039-6257(99)00035-1

[R126] Owsley C, Stalvey BT, Wells J, Sloane ME, McGwin G Jr. 2001. Visual risk factors for crash involvement in older drivers with cataract. Arch. Ophthalmol. 119:881–87 11405840 10.1001/archopht.119.6.881

[R127] Pan F, Swanson WH. 2006. A cortical pooling model of spatial summation for perimetric stimuli. J. Vis. 6:1159–71 17209726 10.1167/6.11.2 PMC3777700

[R128] Paulun VC, Schütz AC, Michel MM, Geisler WS, Gegenfurtner KR. 2015. Visual search under scotopic lighting conditions. Vis. Res. 113:155–68 25988753 10.1016/j.visres.2015.05.004 PMC5645078

[R129] Pelli DG, Bex P. 2013. Measuring contrast sensitivity. Vis. Res. 90:10–14 23643905 10.1016/j.visres.2013.04.015 PMC3744596

[R130] Pelli DG, Palomares M, Majaj NJ. 2004. Crowding is unlike ordinary masking: distinguishing feature integration from detection. J. Vis. 4:1136–69 15669917 10.1167/4.12.12

[R131] Pelli DG, Tillman KA. 2008. The uncrowded window of object recognition. Nat. Neurosci. 11:1129–35 18828191 10.1038/nn.2187 PMC2772078

[R132] Pelli DG, Waugh SJ, Martelli M, Crutch SJ, Primativo S, 2016. A clinical test for visual crowding. F1000Research 5:81

[R133] Piper H 1903. Uber die abhangigkeit des reizwertes leuchtender objekte von ihrer flachen-bezsw. Winkelgrosse. Z. Psychol. Physiol. Sinnesorgane 32:98–112

[R134] Pons C, Mazade R, Jin J, Dul MW, Zaidi Q, Alonso J-M. 2017. Neuronal mechanisms underlying differences in spatial resolution between darks and lights in human vision. J. Vis. 17:5 10.1167/17.14.5 PMC5713488 29196762

[R135] Quigley HA, Addicks EM. 1980. Chronic experimental glaucoma in primates. II. Effect of extended intraocular pressure elevation on optic nerve head and axonal transport. Investig. Ophthalmol. Vis. Sci. 19:137–52 6153173

[R136] Quigley HA, Dunkelberger GR, Green WR. 1989. Retinal ganglion cell atrophy correlated with automated perimetry in human eyes with glaucoma. Am. J. Ophthalmol. 107:453–64 2712129 10.1016/0002-9394(89)90488-1

[R137] Ramulu PY, Swenor BK, Jefferys JL, Friedman DS, Rubin GS. 2013. Difficulty with out-loud and silent reading in glaucoma. Investig. Ophthalmol. Vis. Sci. 54:666–72 23074207 10.1167/iovs.12-10618 PMC3559070

[R138] Ramulu PY, West SK, Munoz B, Jampel HD, Friedman DS. 2009. Glaucoma and reading speed: the Salisbury Eye Evaluation project. Arch. Ophthalmol. 127:82–87 19139345 10.1001/archophthalmol.2008.523 PMC2728116

[R139] Ratliff CP, Borghuis BG, Kao YH, Sterling P, Balasubramanian V. 2010. Retina is structured to process an excess of darkness in natural scenes. PNAS 107:17368–73 20855627 10.1073/pnas.1005846107 PMC2951394

[R140] Raza AS, Hood DC. 2015. Evaluation of the structure-function relationship in glaucoma using a novel method for estimating the number of retinal ganglion cells in the human retina. Investig. Ophthalmol. Vis. Sci. 56:5548–56 26305526 10.1167/iovs.14-16366 PMC4553929

[R141] Redmond T, Garway-Heath DF, Zlatkova MB, Anderson RS. 2010a. Sensitivity loss in early glaucoma can be mapped to an enlargement of the area of complete spatial summation. Investig. Ophthalmol. Vis. Sci. 51:6540–48 20671278 10.1167/iovs.10-5718

[R142] Redmond T, Zlatkova MB, Garway-Heath DF, Anderson RS. 2010b. The effect of age on the area of complete spatial summation for chromatic and achromatic stimuli. Investig. Ophthalmol. Vis. Sci. 51:6533–39 20671282 10.1167/iovs.10-5717

[R143] Redmond T, Zlatkova MB, Vassilev A, Garway-Heath DF, Anderson RS. 2013. Changes in Ricco’s area with background luminance in the S-cone pathway. Optom. Vis. Sci. 90:66–74 23241826 10.1097/OPX.0b013e318278fc2b

[R144] Riccò A 1877. Relazione fra il minimo angolo visuale e l’intensità luminosa. Mem. R. Acad. Sci. Lett. Arti. Modena 17:47–160

[R145] Roux-Sibilon A, Rutgé F, Aptel F, Attye A, Guyader N, 2018. Scene and human face recognition in the central vision of patients with glaucoma. PLOS ONE 13:e0193465 29481572 10.1371/journal.pone.0193465 PMC5826536

[R146] Rovamo J 1978.Receptive field density of retinal ganglion cells and cortical magnification factor in man. Med. Biol. 56:97–102 661404

[R147] Rovamo J, Virsu V. 1979. An estimation and application of the human cortical magnification factor. Exp. Brain Res. 37:495–510 520439 10.1007/BF00236819

[R148] Sanes JR, Masland RH. 2015. The types of retinal ganglion cells: current status and implications for neuronal classification. Annu. Rev. Neurosci. 38:221–46 25897874 10.1146/annurev-neuro-071714-034120

[R149] Schefrin BE, Bieber ML, McLean R, Werner JS. 1998. The area of complete scotopic spatial summation enlarges with age. J. Opt. Soc. Am. A 15:340–48 10.1364/josaa.15.000340 9457792

[R150] Schotter ER, Angele B, Rayner K. 2012. Parafoveal processing in reading. Atten. Percept. Psychophys. 74:5–35 22042596 10.3758/s13414-011-0219-2

[R151] Sekuler R, Ball K. 1986. Visual localization: age and practice. J. Opt. Soc. Am. A 3:864–67 3734925 10.1364/josaa.3.000864

[R152] Sellés-Navarro I, Villegas-Pérez MP, Salvador-Silva M, Ruiz-Gómez JM, Vidal-Sanz M. 1996. Retinal ganglion cell death after different transient periods of pressure-induced ischemia and survival intervals. A quantitative in vivo study. Investig. Ophthalmol. Vis. Sci. 37:2002–14 8814140

[R153] Shakarchi AF, Mihailovic A, West SK, Friedman DS, Ramulu PY. 2019. Vision parameters most important to functionality in glaucoma. Investig. Ophthalmol. Vis. Sci. 60:4556–63 31675073 10.1167/iovs.19-28023 PMC6827423

[R154] Shamsi F, Chen V, Liu R, Pergher V, Kwon M. 2021. Functional field of view determined by crowding, aging, or glaucoma under divided attention. Transl. Vis. Sci. Technol. 10:14 10.1167/tvst.10.14.14 PMC8684310 34910102

[R155] Shamsi F, Liu R, Kwon M. 2022a. Binocularly asymmetric crowding in glaucoma and a lack of binocular summation in crowding. Investig. Ophthalmol. Vis. Sci. 63:36 10.1167/iovs.63.1.36 PMC8802025 35084432

[R156] Shamsi F, Liu R, Owsley C, Kwon M. 2022b. Identifying the retinal layers linked to human contrast sensitivity via deep learning. Investig. Ophthalmol. Vis. Sci. 63:27 10.1167/iovs.63.2.27 PMC8859491 35179554

[R157] Smith MA. 2006. Surround suppression in the early visual system. J. Neurosci. 26:3624–25 16597714 10.1523/JNEUROSCI.0236-06.2006 PMC6674136

[R158] Smith ND, Crabb DP, Garway-Heath DF. 2011. An exploratory study of visual search performance in glaucoma. Ophthalmic Physiol. Opt. 31:225–32 21470272 10.1111/j.1475-1313.2011.00836.x

[R159] Smith ND, Glen FC, Monter VM, Crabb DP. 2014. Using eye tracking to assess reading performance in patients with glaucoma: a within-person study. J. Ophthalmol. 2014:120528 24883203 10.1155/2014/120528 PMC4026991

[R160] Sohail M, Hirji SH, Liebmann JM, Glass LD, Blumberg DM. 2023. Remote contrast sensitivity testing seems to correlate with the degree of glaucomatous macular damage. J. Glaucoma 32:533–39 36897654 10.1097/IJG.0000000000002205

[R161] Stamper RL. 1984. The effect of glaucoma on central visual function. Trans. Am. Ophthalmol. Soc. 82:792–826 6398938 PMC1298679

[R162] Stamper RL. 1989. Psychophysical changes in glaucoma. Surv. Ophthalmol. 33(Suppl):309–18 2655144

[R163] Stievenard A, Rouland JF, Peyrin C, Warniez A, Boucart M. 2021. Sensitivity to central crowding for faces in patients with glaucoma. J. Glaucoma 30:140–47 33074958 10.1097/IJG.0000000000001710

[R164] Stringham J, Jammal AA, Mariottoni EB, Estrela T, Urata C, 2020. Visual crowding in glaucoma: structural and functional relationships. Investig. Ophthalmol. Vis. Sci. 61:3214

[R165] Sullivan TJ, De Sa VR. 2006. A model of surround suppression through cortical feedback. Neural Netw. 19:564–72 16500076 10.1016/j.neunet.2005.12.003

[R166] Takagi ST, Kita Y, Yagi F, Tomita G. 2012. Macular retinal ganglion cell complex damage in the apparently normal visual field of glaucomatous eyes with hemifield defects. J. Glaucoma 21:318–25 21423034 10.1097/IJG.0b013e31820d7e9d

[R167] Tan O, Chopra V, Lu AT, Schuman JS, Ishikawa H, 2009. Detection of macular ganglion cell loss in glaucoma by Fourier-domain optical coherence tomography. Ophthalmology 116:2305–14.e1–2 19744726 10.1016/j.ophtha.2009.05.025 PMC2787911

[R168] Tan O, Li G, Lu AT, Varma R, Huang D. 2008. Mapping of macular substructures with optical coherence tomography for glaucoma diagnosis. Ophthalmology 115:949–56 17981334 10.1016/j.ophtha.2007.08.011 PMC2692598

[R169] Tatham AJ, Boer ER, Rosen PN, Della Penna M, Meira-Freitas D, 2014. Glaucomatous retinal nerve fiber layer thickness loss is associated with slower reaction times under a divided attention task. Am. J. Ophthalmol. 158:1008–17 25068641 10.1016/j.ajo.2014.07.028 PMC4515218

[R170] Toet A, Levi DM. 1992. The two-dimensional shape of spatial interaction zones in the parafovea. Vis. Res. 32:1349–57 1455707 10.1016/0042-6989(92)90227-a

[R171] Turano R 1999. Mobility performance in glaucoma. Investig. Ophthalmol. Vis. Sci. 40:2803–9 10549639

[R172] Turner MH, Schwartz GW, Rieke F. 2018. Receptive field center-surround interactions mediate context-dependent spatial contrast encoding in the retina. eLife 7:e38841 30188320 10.7554/eLife.38841 PMC6185113

[R173] Vasilev A, Anderson RS, Zlatkova M. 2003. Invariants of spatial summation for S (short wavelength) cone vision. Ross. Fiziol. Zh. Im. I M Sechenova 89:1250–57 14758649

[R174] Vassilev A, Ivanov I, Zlatkova MB, Anderson RS. 2005. Human S-cone vision: relationship between perceptive field and ganglion cell dendritic field. J. Vis. 5:823–33 16441188 10.1167/5.10.6

[R175] Vassilev A, Mihaylova MS, Racheva K, Zlatkova M, Anderson RS. 2003. Spatial summation of S-cone ON and OFF signals: effects of retinal eccentricity. Vis. Res. 43:2875–84 14568375 10.1016/j.visres.2003.08.002

[R176] Viswanathan AC, McNaught AI, Poinoosawmy D, Fontana L, Crabb DP, 1999. Severity and stability of glaucoma: patient perception compared with objective measurement. Arch. Ophthalmol. 117:450–54 10206571 10.1001/archopht.117.4.450

[R177] Vlasiuk A, Asari H. 2021. Feedback from retinal ganglion cells to the inner retina. PLOS ONE 16:e0254611 34292988 10.1371/journal.pone.0254611 PMC8297895

[R178] Volbrecht VJ, Shrago EE, Schefrin BE, Werner JS. 2000a. Ricco’s areas for S- and L-cone mechanisms across the retina. Color Res. Appl. 26:S32–35 19763239 10.1002/1520-6378(2001)26:1+<::AID-COL8>3.0.CO;2-V PMC2745110

[R179] Volbrecht VJ, Shrago EE, Schefrin BE, Werner JS. 2000b. Spatial summation in human cone mechanisms from 0 degrees to 20 degrees in the superior retina. J. Opt. Soc. Am. A 17:641–50 10.1364/josaa.17.000641 PMC2698456 10708046

[R180] Wall M, Lefante J, Conway M. 1991. Variability of high-pass resolution perimetry in normals and patients with idiopathic intracranial hypertension. Investig. Ophthalmol. Vis. Sci. 32:3091–95 1938283

[R181] Wang DL, Raza AS, de Moraes CG, Chen M, Alhadeff P, 2015. Central glaucomatous damage of the macula can be overlooked by conventional OCT retinal nerve fiber layer thickness analyses. Transl. Vis. Sci. Technol. 4:4 10.1167/tvst.4.6.4 PMC4669632 26644964

[R182] Wang M, Hood DC, Cho JS, Ghadiali Q, de Moraes CG, 2009. Measurement of local retinal ganglion cell layer thickness in patients with glaucoma using frequency-domain optical coherence tomography. Arch. Ophthalmol. 127:875–81 19597108 10.1001/archophthalmol.2009.145 PMC2987580

[R183] Wässle H, Grünert U, Röhrenbeck J, Boycott BB. 1990. Retinal ganglion cell density and cortical magnification factor in the primate. Vis. Res. 30:1897–911 2288097 10.1016/0042-6989(90)90166-i

[R184] Watson AB. 1987. Estimation of local spatial scale. J. Opt. Soc. Am. A 4:1579–82 3625339 10.1364/josaa.4.001579

[R185] Wiecek E, Pasquale LR, Fiser J, Dakin S, Bex PJ. 2012. Effects of peripheral visual field loss on eye movements during visual search. Front. Psychol. 3:472 23162511 10.3389/fpsyg.2012.00472 PMC3498877

[R186] Wilensky JT, Hawkins A. 2001. Comparison of contrast sensitivity, visual acuity, and Humphrey visual field testing in patients with glaucoma. Trans. Am. Ophthalmol. Soc. 99:213–17; discussion 217–18 11797309 PMC1359012

[R187] Wilson ME. 1970. Invariant features of spatial summation with changing locus in the visual field. J. Physiol. 207:611–22 5499738 10.1113/jphysiol.1970.sp009083 PMC1348730

[R188] Wolfe B, Dobres J, Rosenholtz R, Reimer B. 2017. More than the useful field: considering peripheral vision in driving. Appl. Ergonom. 65:316–25 10.1016/j.apergo.2017.07.009 28802451

[R189] Wolter JR. 1972. The visual fields: a textbook and atlas of clinical perimetry. J. Pediatr. Ophthalmol. 9:190

[R190] Wood JM, Black AA, Mallon K, Thomas R, Owsley C. 2016. Glaucoma and driving: on-road driving characteristics. PLOS ONE 11:e0158318 27472221 10.1371/journal.pone.0158318 PMC4966939

[R191] Wood JM, Owsley C. 2014. Useful field of view test. Gerontology 60:315–18 24642933 10.1159/000356753 PMC4410269

[R192] Xiong Y-Z, Kwon M, Bittner AK, Virgili G, Giacomelli G, Legge GE. 2020. Relationship between acuity and contrast sensitivity: differences due to eye disease. Investig. Ophthalmol. Vis. Sci. 61:40 10.1167/iovs.61.6.40 PMC7415312 38755787

[R193] Yantis S 2014. Sensation and Perception. New York: Worth Publ.

[R194] Zaghloul KA, Boahen K, Demb JB. 2003. Different circuits for ON and OFF retinal ganglion cells cause different contrast sensitivities. J. Neurosci. 23:2645–54 12684450 10.1523/JNEUROSCI.23-07-02645.2003 PMC6742092

[R195] Zhang C, Tatham AJ, Weinreb RN, Zangwill LM, Yang Z, 2014. Relationship between ganglion cell layer thickness and estimated retinal ganglion cell counts in the glaucomatous macula. Ophthalmology 121:2371–79 25148790 10.1016/j.ophtha.2014.06.047 PMC4252259

[R196] Zwierko T, Jedziniak W, Florkiewicz B, Ceylan Hİ, Lesiakowski P, 2020. The consequences of glaucoma on mobility and balance control in the older adults: a cross-sectional study. J. Aging Phys. Act. 29:372–81 32994380 10.1123/japa.2020-0079

[R197] Zwierko T, Jedziniak W, Lesiakowski P, Śliwiak M, Kirkiewicz M, Lubiński W. 2019. Eye-hand coordination impairment in glaucoma patients. Int. J. Environ. Res. Public Health 16:4332 31703245 10.3390/ijerph16224332 PMC6888341

